# Oxide-ion conduction in the Dion–Jacobson phase CsBi_2_Ti_2_NbO_10*−δ*_

**DOI:** 10.1038/s41467-020-15043-z

**Published:** 2020-03-06

**Authors:** Wenrui Zhang, Kotaro Fujii, Eiki Niwa, Masato Hagihala, Takashi Kamiyama, Masatomo Yashima

**Affiliations:** 1 Department of Chemistry, School of Science, Tokyo Institute of Technology, 2–12–1 W4–17 O-okayama, Meguro-ku, Tokyo, 152–8551 Japan; 2 Institute of Materials Structure Science, High Energy Accelerator Research Organization (KEK), 203-1 Tokai, Ibaraki, 319–1106 Japan

**Keywords:** Materials chemistry, Fuel cells, Structure of solids and liquids, Fuel cells, Structure of solids and liquids

## Abstract

Oxide-ion conductors have found applications in various electrochemical devices, such as solid-oxide fuel cells, gas sensors, and separation membranes. Dion–Jacobson phases are known for their rich magnetic and electrical properties; however, there have been no reports on oxide-ion conduction in this family of materials. Here, for the first time to the best of our knowledge, we show the observation of fast oxygen anionic conducting behavior in CsBi_2_Ti_2_NbO_10−*δ*_. The bulk ionic conductivity of this Dion–Jacobson phase is 8.9 × 10^−2^ S cm^−1^ at 1073 K, a level that is higher than that of the conventional yttria-stabilized zirconia. The oxygen ion transport is attributable to the large anisotropic thermal motions of oxygen atoms, the presence of oxygen vacancies, and the formation of oxide-ion conducting layers in the crystal structure. The present finding of high oxide-ion conductivity in rare-earth-free CsBi_2_Ti_2_NbO_10−*δ*_ suggests the potential of Dion–Jacobson phases as a platform to identify superior oxide-ion conductors.

## Abstract (web summary)

Oxide ion conductors are an exciting class of materials with applications in various domains. Here, the authors show that Dion–Jacobson Phases are a structure supporting high O^2−^ mobility. The bulk conductivity of CsBi_2_Ti_2_NbO_10−*δ*_ even exceeds that of YSZ, offering new possibilities in electrolyte discovery.

## Introduction

Oxide-ion conductors have attracted much attention because of their extensive applications, including in solid-oxide fuel cells, gas sensors, oxygen separation membranes, and catalysts^1–9^. Due to the interaction of oxide ions with the cation network, high oxide-ion conductivities have been achieved in a limited number of structure families; for example, the fluorite-type, perovskite-type, melilite-type, and apatite-type structures^10–17^. Since the limited structure families restrict further development in chemistry and solid-state ionics, the discovery of oxide-ion conductors with new crystal structures is of vital importance for the development of their applications. Various layered perovskites such as BIMEVOX^18–21^, the Aurivillius phase^22–24^, the Ruddlesden–Popper phase^14,15,25–29^, the double perovskites^30–32^, the brownmillerites^33,34^, the hexagonal perovskite derivative (Ba_3_MoNbO_8.5_)^35–37^, and the BaNdInO_4_-based oxides^16,38–40^ were reported to exhibit high oxide-ion conductivities. The Dion–Jacobson phase is an *A*′/*A* cation-ordered layered perovskite with a general formula of *A*′ [*A*_*n*−1_*B*_*n*_O_3*n*+1_] (*A*′ = Cs, Rb, Li, H, Ag; *A* = La, Ca, Sr, Bi; and *B* = Ti, Nb, Ta, etc.), where *n* denotes the number of the *B*O_6_ octahedral layers, and the *A*′ cation separates the *A*_*n*−1_*B*_*n*_O_3*n*+1_ perovskite-like layers^41,42^. Thus, oxide-ion conduction can be expected in the Dion–Jacobson phases. Numerous studies have been conducted on the electrical properties of the Dion–Jacobson phases, such as ferroelectricity^43^, proton^44,45^, lithium-ion^46,47^, sodium-ion^47,48^, and mixed proton–electron conduction^49^. However, there are no reports on oxide-ion conduction in the Dion–Jacobson phases. Herein, we report oxide-ion conduction in the Dion–Jacobson phase for the first time.

Here, we have screened 69 Dion–Jacobson phases using available crystallographic data and the bond-valence method (see the details in Supplementary Methods, Supplementary Fig. 1 and Supplementary Table 1)^12,13,16,17,37,38,40,50–53^. The chemical composition of CsBi_2_Ti_2_NbO_10_ is selected because the bond-valence-based energy barrier for the oxide-ion migration, *E*_b_, is relatively low (*E*_b_ = 0.5 eV) and CsBi_2_Ti_2_NbO_10_ does not contain expensive rare-earth elements. Surprisingly, it is found that the bulk conductivities (*σ*_b_) of the Dion–Jacobson phase of CsBi_2_Ti_2_NbO_10−*δ*_ are as high as 8.9 × 10^−2^ S cm^−1^ at 1073 K and 1.5 × 10^−2^ S cm^−1^ at 873 K, which are higher than those of the conventional yttria-stabilized zirconia (YSZ). In consideration of the wide compositional space in Dion–Jacobson phases *A*′ [*A*_*n*−1_*B*_*n*_O_3*n*+1_] (where *A*′ = Cs, Rb, Li, H, Ag; *A* = La, Ca, Sr, Bi; *B* = Ti, Nb, Ta, etc., and *n* ranges from 2 to 6, and examples are presented in Supplementary Table 1), the present discovery provides new possibilities in the chemistry of oxide-ion conductors.

## Results

### Phase transition and oxygen content of CsBi_2_Ti_2_NbO_10−*δ*_

**Fig. 1:**
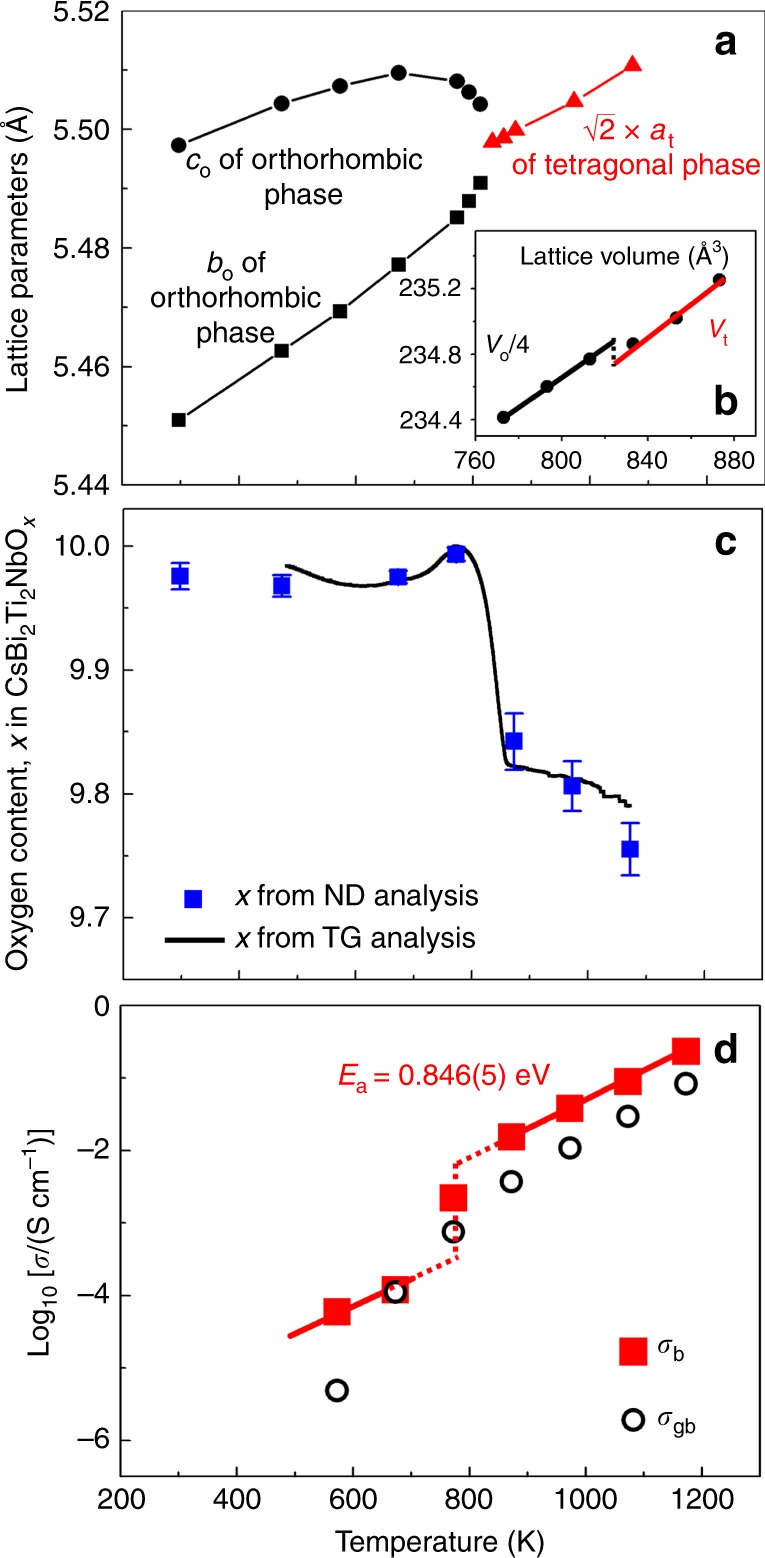
Temperature dependences of lattice parameters, oxygen content, and conductivities. Lattice parameters (**a**) and reduced lattice volume (**b**) of CsBi_2_Ti_2_NbO_10−*δ*_, which were refined using synchrotron X-ray powder diffraction data measured in situ at high temperatures in static air on heating. The subscripts o and t denote orthorhombic and tetragonal, respectively. **c** Oxygen contents of CsBi_2_Ti_2_NbO_10−*δ*_ on heating, which were obtained by thermogravimetric (TG) analysis in dry air (black line) and calculated from occupancy factors refined using in situ neutron-diffraction (ND) data in vacuum (blue marks). **d** Bulk electrical conductivity *σ*_b_ and grain boundary conductivity *σ*_gb_ of CsBi_2_Ti_2_NbO_10−*δ*_ in dry air on heating.

CsBi_2_Ti_2_NbO_10−*δ*_ was synthesized by the solid-state reactions. The Rietveld refinements of the synchrotron X-ray diffraction data taken in static air and neutron-diffraction data obtained in vacuum were successfully performed using a single orthorhombic *Ima*2 structure at 297–813 K and a single tetragonal *P*4/*mmm* structure at 833–1073 K on heating (Fig. 1a, Supplementary Figs. 2, 3a, 4a, 5, and Supplementary Tables 2 and 3). The 002 and 020 peaks and 602 and 620 ones of the orthorhombic phase approached each other and merged at a temperature between 813 and 833 K on heating (Supplementary Fig. 4a). The *b-* and *c*-axis lengths of the orthorhombic phase continuously approached each other, leading to thermal expansion anomalies (Supplementary Fig. 6), and coincided at a temperature between 813 and 833 K on heating (Fig. 1a and Supplementary Fig. 3b). These results indicate that the orthorhombic-to-tetragonal (o-to-t) phase transition occurred at a temperature between 813 and 833 K on heating, which is consistent with the literature^43^. The reversible t-to-o transition was observed at a temperature between 793 and 813 K on cooling (Supplementary Fig. 4b), exhibiting a hysteresis of about 20 K. In addition, the reduced lattice volume of CsBi_2_Ti_2_NbO_10−*δ*_ discontinuously decreased between the orthorhombic and tetragonal phases (the lattice volume change is −0.042(5)%) (Fig. 1b). The hysteresis and discontinuous decrease indicate that the o-t phase transition is first order. It was found that the o-to-t transition was accompanied by 0.32 wt% weight loss on heating, as shown in the thermogravimetric (TG) results (black line in Fig. 1c). The weight loss was caused by an increase in the oxygen vacancy concentration (blue squares in Fig. 1c). The presence of oxygen vacancies (*δ*) in the high-temperature tetragonal phase of CsBi_2_Ti_2_NbO_10−*δ*_ was confirmed by both the oxygen occupancy factors refined using variable temperature neutron-diffraction data and oxygen contents estimated by the TG analysis (Fig. 1c). The oxygen vacancies are responsible for the high oxide-ion conductivities described later in this paper.

### Oxide-ion conduction of CsBi_2_Ti_2_NbO_10−*δ*_

**Fig. 2:**
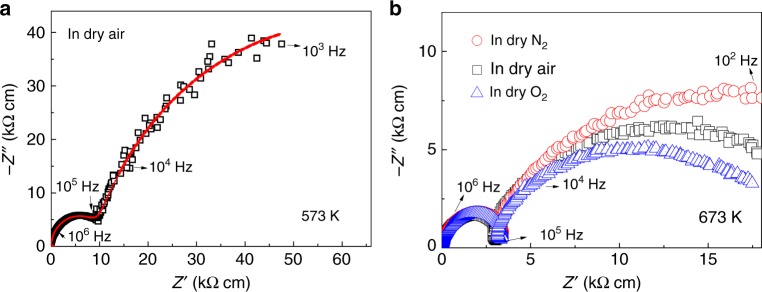
Complex impedance plots of CsBi_2_Ti_2_NbO_10−*δ*_. **a** Complex impedance plots recorded in flowing dry air at 573 K on heating. Red line indicates the equivalent circuit fitting. The equivalent circuit used to model the impedance data and complex impedance plots at different temperatures are shown in Supplementary Fig. 7. **b** Complex impedance plots measured in flowing dry N_2_, air, and O_2_ gases at 673 K on heating.

The electrical conductivities of CsBi_2_Ti_2_NbO_10−*δ*_ were measured from 573 to 1173 K using AC and DC methods. Figure 2 shows the typical impedance spectra of CsBi_2_Ti_2_NbO_10−*δ*_. The bulk and grain boundary responses were observed [respective capacitance values of *C*_b_ ≈ 0.7 × 10^−12^ F cm^−1^ and *C*_gb_ ≈ 7.0 × 10^−9^ F cm^−1^ at 573 K in dry air (Fig. 2a)]. The bulk conductivities (*σ*_b_) were independent of the oxygen partial pressure (dry O_2_, dry air, and dry N_2_; Fig. 2b), which indicates ionic conduction in CsBi_2_Ti_2_NbO_10−*δ*_. Figure 1d shows the temperature dependence of the *σ*_b_ and grain boundary conductivities (*σ*_gb_) of CsBi_2_Ti_2_NbO_10−*δ*_ on heating. The *σ*_b_ abruptly increased between 673 and 873 K on heating, which was attributed to the increase in the carrier (oxygen vacancy) concentration (Fig. 1c) and the o-to-t phase transition (Fig. 1a, b). The total DC electrical conductivity also exhibited an abrupt increase around the o-to-t transition point on heating (Supplementary Fig. 8).

**Fig. 3:**
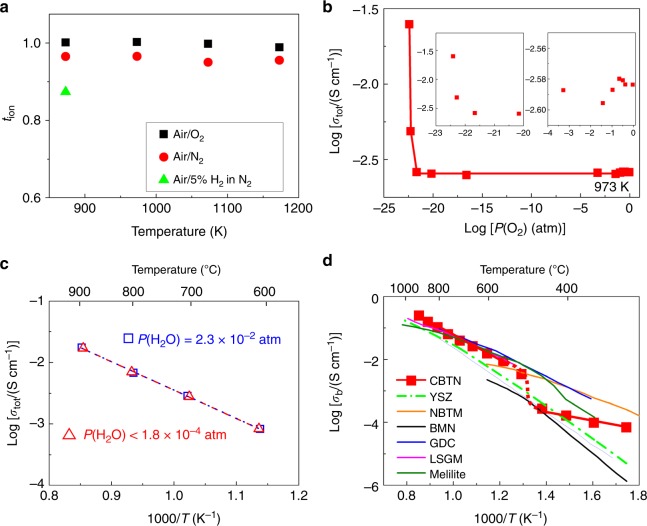
Oxide-ion conduction of CsBi_2_Ti_2_NbO_10−*δ*_. **a** Oxide-ion transport number *t*_ion_ determined by oxygen concentration cell measurements of CsBi_2_Ti_2_NbO_10−*δ*_. **b** Partial oxygen pressure *P*(O_2_) dependence of total DC electrical conductivities *σ*_tot_ of CsBi_2_Ti_2_NbO_10−*δ*_ at 973 K and the insets show the zoom-in view of high and low *P*(O_2_) regions. **c** Arrhenius plots of *σ*_tot_ of CsBi_2_Ti_2_NbO_10−*δ*_ in dry and wet air. **d** Comparison of bulk conductivity *σ*_b_ of CsBi_2_Ti_2_NbO_10−*δ*_ (CBTN) on cooling with those of best oxide-ion conductors: (Y_2_O_3_)_0.08_(ZrO_2_)_0.92_ (YSZ)^54^, Na_0.5_Bi_0.49_Ti_0.98_Mg_0.02_O_2.965_ (NBTM)^55^, Ba_3_MoNbO_8.5_ (BMN)^36^, Ce_0.9_Gd_0.1_O_1.95_ (GDC)^56^, La_0.9_Sr_0.1_Ga_0.8_Mg_0.2_O_2.85_ (LSGM)^6^, and La_1.54_Sr_0.46_Ga_3_O_7.27_ (melilite)^57^.

Oxygen concentration cell measurements were performed to determine the oxide-ion transport number (*t*_ion_). The *t*_ion_ values were 1.00–0.98 between 873 and 1173 K in air/O_2_, 0.97–0.95 between 873 and 1173 K in air/N_2_, and 0.87 at 873 K in air/5% H_2_ in N_2_ (Fig. 3a). The total DC electrical conductivity (*σ*_tot_) at 973 K was almost independent of the oxygen partial pressure *P*(O_2_) between *P*(O_2_) = 2.0 × 10^−22^ and 1 atm (Fig. 3b). At the *P*(O_2_) < 2.0 × 10^−22^ region, an n-type electronic contribution to the total conductivity was observed, which is consistent with the relatively low *t*_ion_ in air/5% H_2_ in N_2_. The electronic conduction is attributable to the formation of oxygen vacancies and electronic defects accompanied by reduction of Ti^4+^ and/or Nb^5+^ cations in the reducing atmosphere. No significant proton conduction was observed between 873 and 1173 K because the conductivities measured in dry air (H_2_O partial pressure, *P*(H_2_O) < 1.8 × 10^−4^ atm) agreed well with those in wet air (*P*(H_2_O) = 2.3 × 10^−2^ atm) (Fig. 3c). These results indicate that the oxide ion is the dominant carrier and that CsBi_2_Ti_2_NbO_10−*δ*_ is an oxide-ion conductor. No change was observed in the X-ray powder diffraction patterns before and after the impedance spectroscopy and oxygen concentration cell measurements (Supplementary Fig. 9), which demonstrated the high phase stability of CsBi_2_Ti_2_NbO_10−*δ*_ at high temperatures and different *P*(O_2_) values. The *σ*_b_ of CsBi_2_Ti_2_NbO_10−*δ*_ was higher than that of YSZ and comparable with those of the best oxide-ion conductors (Fig. 3d). This indicates the high potential of the Dion–Jacobson phase CsBi_2_Ti_2_NbO_10−*δ*_ as a basic composition for oxide-ion conductors.

### Structural origin of the high oxide-ion conductivity of CsBi_2_Ti_2_NbO_10−*δ*_

**Fig. 4:**
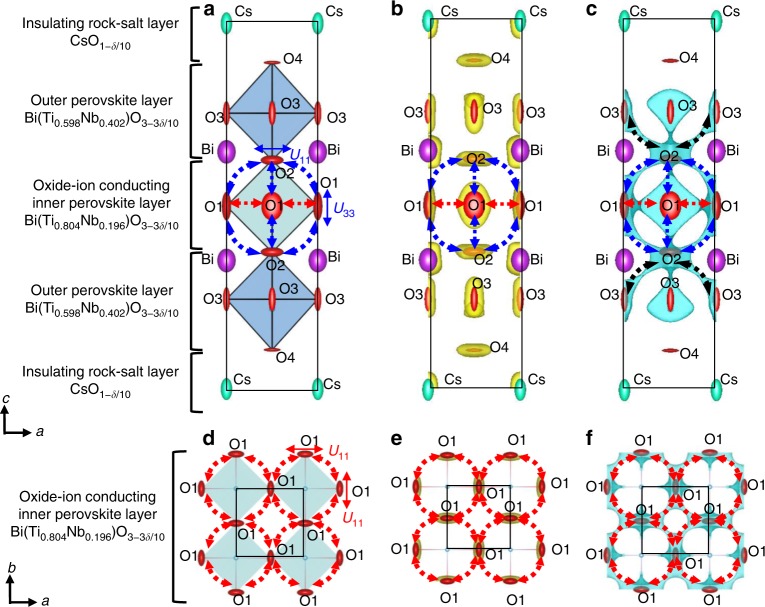
Crystal structure and oxide-ion diffusion pathway of tetragonal CsBi_2_Ti_2_NbO_9.80(2)_ at 973 K. **a**, **d** Refined crystal structure of CsBi_2_Ti_2_NbO_9.80(2)_ at 973 K, which was obtained by Rietveld analysis of in situ neutron-diffraction data. **b**, **e** Yellow isosurfaces of the neutron scattering length density at 1.0 fm Å^−3^ with the structure (973 K). **c**, **f** Blue isosurfaces of the bond-valence-based energy for an oxide ion at 0.6 eV for the structure at 973 K. Blue, red, and black dotted lines with arrows denote the possible O1–O2, O1–O1, and O2–O3 diffusion pathways of oxide ion, respectively. The solid lines with arrows in **a** and **d** stand for the directions of anisotropic thermal motions of O1 and O2 oxygen atoms. Thermal ellipsoids are drawn at the 50% probability level. Regions of **a**–**c** −1/2 ≤ *x*, *y*, *z* ≤ 1/2 and of **d**–**f** −1/2 ≤ *x*, *y* ≤ 3/2, 0 ≤ *z* ≤ 0.1.

Next, we discuss the structural origin of the high oxide-ion conductivity using the crystal structure of tetragonal CsBi_2_Ti_2_NbO_9.80(2)_ at 973 K (Fig. 4). This structure was obtained by the Rietveld refinement of the neutron-diffraction data measured in situ at 973 K with a super-high-resolution diffractometer, SuperHRPD^58,59^. The tetragonal structure of CsBi_2_Ti_2_NbO_10−*δ*_ consists of an oxide-ion conducting inner perovskite Bi-(Ti_0.804_Nb_0.196_)O_3−3*δ*/10_ layer, two outer perovskite Bi-(Ti_0.598_Nb_0.402_)O_3−3*δ*/10_ layers, and an insulating CsO_1−*δ*/10_ rock-salt layer (Fig. 4a and Supplementary Fig. 5). The refined equivalent isotropic atomic displacement parameters of the equatorial oxygen atom, O1, (*U*_eq_(O1)) and apical oxygen, O2, (*U*_eq_(O2)) in the oxide-ion conducting inner perovskite layer were much higher than those of the equatorial O3 and apical O4 atoms in the outer perovskite layers (*U*_eq_(O1) = 0.0879(9) Å^2^, *U*_eq_(O2) = 0.0734(8) Å^2^ >> *U*_eq_(O3) = 0.0441(5) Å^2^, *U*_eq_(O4) = 0.0382(5) Å^2^). The higher atomic displacement parameters of O1 and O2 were consistent with the results in the literature^43^ and from the synchrotron X-ray diffraction data (Supplementary Table 4). Therefore, the O1 and O2 atoms in the inner oxide-ion conducting perovskite layer exhibited larger thermal motions than the O3 and O4 atoms in the outer perovskite layer. In particular, the anisotropic atomic displacement parameters (*U*_*ij*_) of the O1 and O2 atoms were extremely large: *U*_11_(O1) = 0.0901(19) Å^2^, *U*_33_(O1) = 0.157(3) Å^2^, and *U*_11_(O2) = *U*_22_(O2) = 0.1010(11) Å^2^ (see others in Supplementary Table 3). The large *U*_33_(O1) and *U*_11_(O2) values indicate high anisotropic thermal motions along the *c* and *a* axes, respectively, which suggests the O1–O2 oxide-ion diffusion (blue-dotted arrows in Fig. 4a). Similarly, the large *U*_11_(O1) means that there are high thermal motions along the *a* and *b* axes, indicating the O1–O1 oxide-ion diffusion (the red-dotted arrows in Fig. 4d). The isosurfaces of the neutron scattering length density obtained by the maximum-entropy method (MEM)^11,12,14,15,31,37,60,61^ (yellow isosurfaces in Fig. 4b, e) and bond-valence-based energy landscapes (BVELs, blue isosurfaces in Supplementary Fig. 10) also showed anisotropic thermal motions of O1 and O2 atoms. The O1–O2 and O1–O1 oxide-ion diffusion paths were clearly observed in the BVELs (dotted arrows in Fig. 4c, f). In addition, the O2–O3 diffusion path was observed in the BVELs (the black dotted arrows in Fig. 4c). These results indicate two-dimensional (2D) oxide-ion diffusion along the edges of the Bi(Ti_0.804_Nb_0.196_)O_3−3*δ*/10_ octahedron in the oxide-ion conducting inner perovskite layer. We attribute the high oxide-ion conductivity of CsBi_2_Ti_2_NbO_10−*δ*_ not only to the existence of oxygen vacancies but also to the 2D oxide-ion diffusion and the extremely high *U*_eq_(O1), *U*_eq_(O2), *U*_11_(O1), *U*_33_(O1), and *U*_11_(O2) [=*U*_22_(O2)]. In fact, these thermal parameters of CsBi_2_Ti_2_NbO_10−*δ*_ are much higher than those of perovskite-type and layered perovskite-type conductors, such as La_0.9_Sr_0.1_Ga_0.79_Mg_0.21_O_2.82_^62^, La_0.8_Sr_0.2_Ga_0.8_Mg_0.15_Co_0.05_O_2.8_^63^, BaBi_4_Ti_4_O_15_^23^, Bi_4_Ti_3_O_12_^64^, and Bi_2_Sr_2_TiNb_2_O_12_^65^ (Supplementary Tables 5 and 6).

## Discussion

**Fig. 5:**
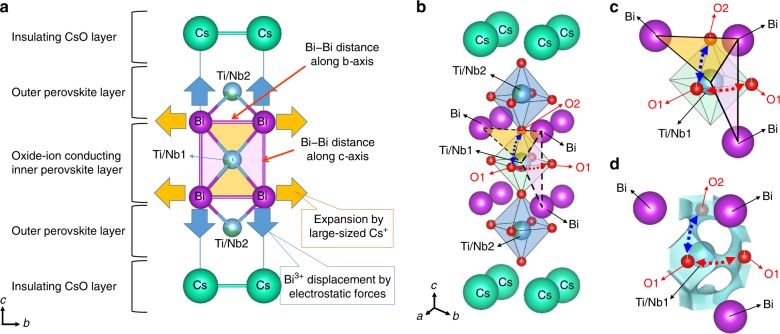
New concept of enlarged bottlenecks for oxide-ion migration by large size of Cs^+^ and Bi^3+^ displacement. **a** New concept of enlarged bottlenecks for oxide-ion migration created by large size of Cs^+^ and Bi^3+^ displacement in Dion–Jacobson-type Cs/Bi-cation-ordered CsBi_2_Ti_2_NbO_10−*δ*_ (−1/2 ≤ *x*, *y*, *z* ≤ 1/2). Yellow and pink Bi–Bi–Ti triangles stand for the areas of bottlenecks for O1–O2 and O1–O1 oxide-ion migration, respectively. Oxygen atoms are omitted for simplicity in **a**. **b** Refined structure and Bi–Bi–Ti/Nb1 triangle bottleneck of CsBi_2_Ti_2_NbO_9.80(2)_ at 973 K (−1/2 ≤ *x*, *y*, *z* ≤ 1/2). A part of the structure (**c**) and corresponding bond-valence-based energy landscape (BVEL) with the isosurface at 0.6 eV (**d**), showing the O1–O2 and O1–O1 oxide-ion diffusion paths. Blue and red-dotted lines with arrows denote the possible O1–O2 and O1–O1 diffusion pathways of oxide ion, respectively.

Here, we propose a new concept: large bottlenecks for oxide-ion migration by the large size of Cs^+^ and Bi^3+^ displacement. The bottlenecks of CsBi_2_Ti_2_NbO_10−*δ*_ are the Bi–Bi–Ti triangles (the yellow and pink triangles in Fig. 5a–c). The large bottlenecks of CsBi_2_Ti_2_NbO_10−*δ*_ can be explained using the new concept, as described below. Cs^+^ expands the Bi–Bi distance along the *b* axis (the orange arrows in Fig. 5a) due to the occupational ordering of the large Cs^+^ and small Bi^3+^ cations. Bi^3+^ is displaced along the *c* axis apart from the Ti/Nb1–O1 layer (the blue arrows in Fig. 5a), by electrostatic forces (Supplementary Note 1 and Supplementary Fig. 11). The Bi^3+^ displacement increases the Bi–Bi distance along the *c* axis. The increase in the Bi–Bi distances along both the *b* (=*a*) and *c* axes leads to large bottlenecks for the O1–O2 (the yellow areas) and O1–O1 (the pink areas) oxide-ion diffusion (Fig. 5b–d***)***. Since the bottleneck of CsBi_2_Ti_2_NbO_10−*δ*_ is the Bi–Bi–Ti triangle, it is interesting to compare the bottleneck sizes (critical radii) and oxide-ion conductivities of CsBi_2_Ti_2_NbO_10−*δ*_ with those of layered perovskites with Bi–Bi–Ti bottleneck triangles, BaBi_4_Ti_4_O_15_, Bi_4_Ti_3_O_12_, and Bi_2_Sr_2_TiNb_2_O_12_. The oxide-ion conductivity of CsBi_2_Ti_2_NbO_10−*δ*_ was much higher than those of these materials, which is attributable to the larger bottlenecks (Supplementary Table 7).

In summary, we have discovered the first example of the Dion–Jacobson-type oxide-ion conductor (CsBi_2_Ti_2_NbO_10−*δ*_). It was found that CsBi_2_Ti_2_NbO_10−*δ*_ exhibits a high *σ*_b_ of 8.9 × 10^−2^ S cm^−1^ at 1073 K and high phase stability at high temperatures and different *P*(O_2_) values. The conductivity abruptly increased between 673 and 873 K on heating, which is ascribed to the increase in oxygen vacancy concentration and the o-to-t phase transition. The high oxide-ion conductivities of Cs/Bi-cation-ordered CsBi_2_Ti_2_NbO_10−*δ*_ are attributable to (i) the large anisotropic thermal motions of the oxygen atoms in the inner oxide-ion conducting perovskite layer, (ii) the 2D O1–O2 and O1–O1 oxide-ion diffusion, (iii) the existence of oxygen vacancies and (iv) the large bottlenecks. We have also proposed a concept: large bottlenecks for oxide-ion migration by large size of Cs^+^ and Bi^3+^ displacement. The oxide-ion conductivity is expected to be improved by doping and/or modification of the chemical composition of the basic material CsBi_2_Ti_2_NbO_10−*δ*_. The present finding of high oxide-ion conductivities in the new structure family, Dion–Jacobson-type CsBi_2_Ti_2_NbO_10−*δ*_, and the concept would facilitate the design of novel oxide-ion conductors based on the Dion–Jacobson phases.

## Methods

### Synthesis and characterization of CsBi_2_Ti_2_NbO_10−*δ*_

CsBi_2_Ti_2_NbO_10−*δ*_ was synthesized by the solid-state-reaction method. High-purity (>99.9%) Cs_2_CO_3_, Bi_2_O_3_, TiO_2_, and Nb_2_O_5_ (with molar ratios of Cs, Bi, Ti, and Nb = 1.3: 2.0: 2.0: 1.0) were mixed and ground using an agate mortar and a pestle for 30 min as ethanol slurries, where an excess amount of Cs_2_CO_3_ (30 mol%) was added to compensate for the mass loss due to the volatilisation of the Cs species during sintering^43^. The mixture was dried on a hot plate (with a setting temperature of 373 K) and then ground into powder in the mortar for 30 min. This mixing, drying, and grinding processes were repeated a few times. The obtained mixtures were uniaxially pressed into pellets at about 100 MPa and subsequently sintered in static air at 1173 K for 12 h at heating and cooling rates of 5 K min^−1^. The grinding, pressing, and sintering processes were repeated a few times until a single phase of CsBi_2_Ti_2_NbO_10−*δ*_ was obtained. Parts of the sintered pellets were crushed and ground into powders to carry out X-ray powder diffraction, synchrotron X-ray powder diffraction, atomic absorption spectroscopy (AAS, Hitachi Z-2300), inductively coupled plasma optical emission spectroscopy (ICP-OES, Hitachi PS3520UVDD), and TG measurements. The existing phase of CsBi_2_Ti_2_NbO_10−*δ*_ was examined with an X-ray powder diffractometer (RINT-2550, Cu *K*α radiation, 2*θ* range: 5–100°, Step interval: 0.02°). The AAS and ICP-OES results indicated that the cation molar ratios of CsBi_2_Ti_2_NbO_10−*δ*_ were Cs: Bi: Ti: Nb = 1: 2: 2: 1, which were consistent with the nominal composition. TG analysis was carried out in flowing dry and static air between 297 and 1073 K using a Bruker-AXS 2020SA instrument at heating and cooling rates of 1 K min^−1^. The measurements between 473 and 1073 K were repeated three times to negate the influence of absorbed species, such as water, and also to confirm the reproducibility. The TG data on the third heating in dry air were plotted in Fig. 1c. The TG data in dry air agreed well with those in static air. The sample did not decompose during the TG measurements, which was confirmed by X-ray powder diffraction measurements of the sample after the TG measurements.

### Structure analyses by high-temperature synchrotron X-ray and neutron diffraction

High-angular-resolution SXRPD data of CsBi_2_Ti_2_NbO_10−*δ*_ were obtained in static air between 297 and 1073 K, using six 1D solid-state detectors at the BL02B2 beamline of SPring-8, Japan^66,67^. A powdered sample of CsBi_2_Ti_2_NbO_10−*δ*_ was loaded into a quartz glass capillary with an inner diameter of 0.1 mm for the SXRPD measurements. The wavelength was determined to be 0.6992620(4) Å using silicon powder (NIST SRM 640c). The absorption correction was performed through the empirical formula given by Rouse and Cooper^68^.

High-temperature neutron-diffraction measurements were carried out in vacuum using a super-high-resolution time-of-flight neutron diffractometer (SuperHRPD) installed at the Materials and Life Science Experimental Facility of J-PARC, Japan^58,59^. The absorption correction was performed using the method given by Rouse and Cooper^68^. The diffraction data were analysed by the Rietveld method using the Z-Rietveld program^69^. Oxygen contents were calculated using the oxygen occupancy factors refined in the Rietveld analyses of the neutron-diffraction data. The calculated oxygen contents 10 − *δ* in CsBi_2_Ti_2_NbO_10−*δ*_ were 9.84(2) at 873 K, 9.80(2) at 973 K, and 9.76(2) at 1073 K, which were consistent with the TG data: 9.839 at 873 K, 9.828 at 973 K, and 9.807 at 1073 K (Fig. 1c).

The neutron scattering length density distribution was investigated using the MEM. The MEM analysis was carried out with computer program, Dysnomia^70^, using the structure factors obtained in the Rietveld refinement of the neutron-diffraction data at 973 K. The MEM calculations were performed with the unit cell divided into 38 × 38 × 156 pixels. The refined crystal structure, MEM neutron scattering length density distributions, and bond-valence-based energy landscape were depicted by VESTA^71^.

### Conductivity measurements and oxygen concentration cell measurements

The electrical conductivities of CsBi_2_Ti_2_NbO_10−*δ*_ were measured by AC impedance spectroscopy in flowing dry air, N_2_, and O_2_ gases using a sintered pellet (20 mm in diameter, 2.6 mm in thickness, relative density of 91%) with Au electrodes in the temperature range of 573–1173 K on heating and cooling. The surface of the pellet was polished before smearing Au paste to decrease the electrode resistance. Impedance spectra were recorded with a Solartron 1260 impedance analyzer in the frequency range of 15 MHz–1 Hz at an applied alternating voltage of 100 mV. The bulk and grain boundary responses were evidenced, and equivalent circuit analyses were carried out to extract the *σ*_b_ and *σ*_gb_ at each temperature using the ZView software (Scribner Associates, Inc.). The temperature dependence of *σ*_tot_ of CsBi_2_Ti_2_NbO_10−*δ*_ was also measured in dry and wet air on cooling by a DC-4-probe method (4.5 mm in diameter, 11 mm in height, and relative density of 91%). The *P*(O_2_) dependence of the *σ*_tot_ was measured at 973 K in the *P*(O_2_) region between 3 × 10^−23^ and 1 atm using a mixture of O_2_, N_2_, and 5% H_2_ in N_2_ by the DC 4-probe method (4.6 mm in diameter, 12 mm in height, and relative density of 88%). *P*(O_2_) was monitored by an oxygen sensor.

Oxygen concentration cell measurements were performed to determine the oxide-ion transport number *t*_ion_ using a sintered pellet (20 mm in diameter, 4.5 mm in height, and relative density of 91%) attached to an alumina tube with a glass seal. One side of the pellet was exposed to a flowing dry air and the other side to a flowing dry O_2_ (Air/O_2_), N_2_ (Air/N_2_), and 5% H_2_ in N_2_ (Air/5% H_2_ in N_2_) gases between 873 and 1173 K. The electromotive forces of the concentration cell were measured using a Keithley model 617 electrometer. The following Nernst equation was used to estimate the *t*_ion_:

1 $$\begin{array}{*{20}{c}} {E = t_{{\mathrm{ion}}}\frac{{RT}}{{4F}}ln\left( {\frac{{P\left( {{\mathrm{O}}_2} \right)}}{{P^0\left( {{\mathrm{O}}_2} \right)}}} \right)} \end{array}\!,$$

where *F* is the Faraday constant, *R* is the gas constant, *T* is the absolute temperature, *P*(O_2_) is the oxygen partial pressure of the gas of O_2_, N_2_, 5% H_2_ in N_2_, and *P*^0^(O_2_) (=0.21 atm) is the oxygen partial pressure of dry air.

The activation energies, *E*_a_, for the conductivities were estimated using the Arrhenius equation:

2 $$\begin{array}{*{20}{c}} {\sigma = \frac{{A_0}}{T}\exp \left( { - \frac{{E_{\mathrm{a}}}}{{kT}}} \right)} \end{array}\!,$$

where *A*_0_, *k*, and *T* are the pre-exponential factor, Boltzmann constant, and absolute temperature, respectively.

After the impedance spectroscopy and oxygen concentration cell measurements, the surface of the pellet was ground with sandpaper carefully to remove the Au paste and then crushed and ground into powder. The powder was used for the X-ray diffraction measurements to investigate the phase stability at high temperatures and different atmospheres.

## Supplementary information

Supplementary Information

## Data Availability

The data that support the findings of this study are available from the corresponding author upon reasonable request.
