# Supplementary material for: Oxide-ion conduction in the Dion–Jacobson phase CsBi2Ti2NbO10−δ
Source: Nat Commun. 2020 Mar 6;11:1224. doi: 10.1038/s41467-020-15043-z (PMC7060205; doi:10.1038/s41467-020-15043-z)
Supplement: Supplementary file 1 — Supplementary Information [file 41467_2020_15043_MOESM1_ESM.pdf]

Supplementary information

Oxide-Ion Conduction in the Dion–Jacobson Phase $CsBi_2Ti_2NbO_{10-\delta}$

Zhang, W. et al

Supplementary Figures

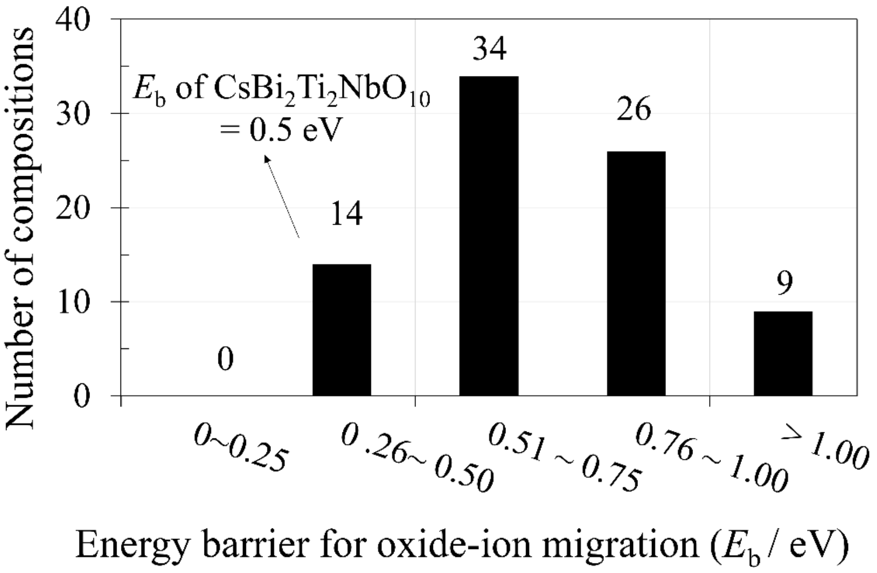

Supplementary Figure 1. Histogram of the bond-valence-based energy barriers E_b for the oxide-ion migration of the 69 Dion−Jacobson phases using 83 crystallographic data.

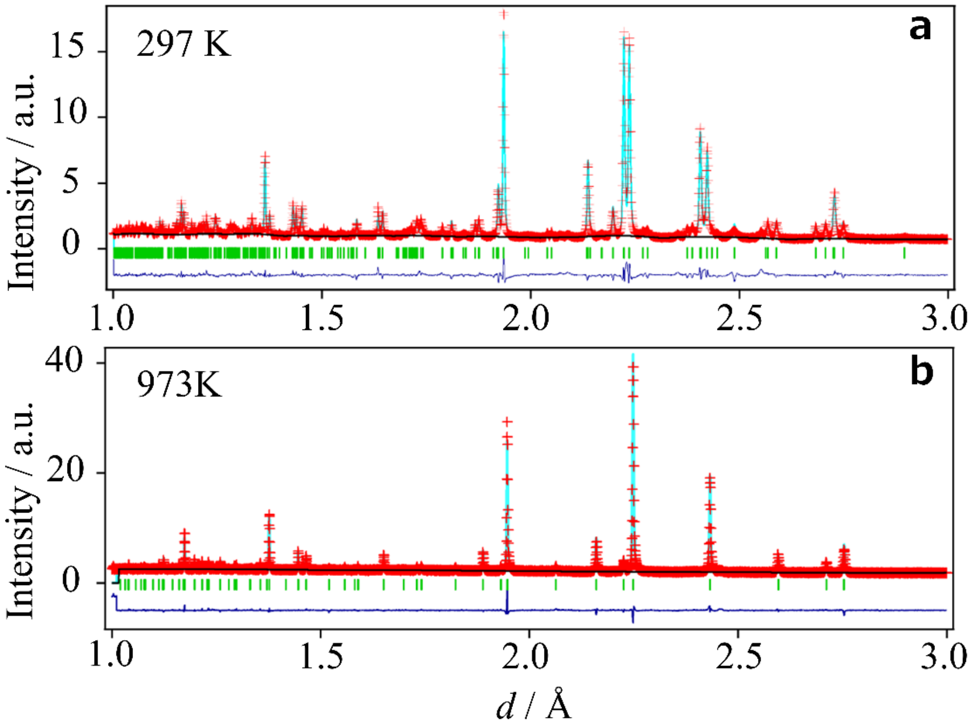

Supplementary Figure 2. Rietveld patterns of $CsBi_2Ti_2NbO_{10-\delta}$. Rietveld patterns at 297 K (**a**) and 973 K (**b**) of neutron-diffraction data taken by the backscattering bank (d = 1.0–3.0 Å) of the SuperHRPD diffractometer. The observed and calculated intensities and difference plots are shown by red marks, light blue and blue solid lines, respectively. Green tick marks stand for calculated Bragg peak positions.

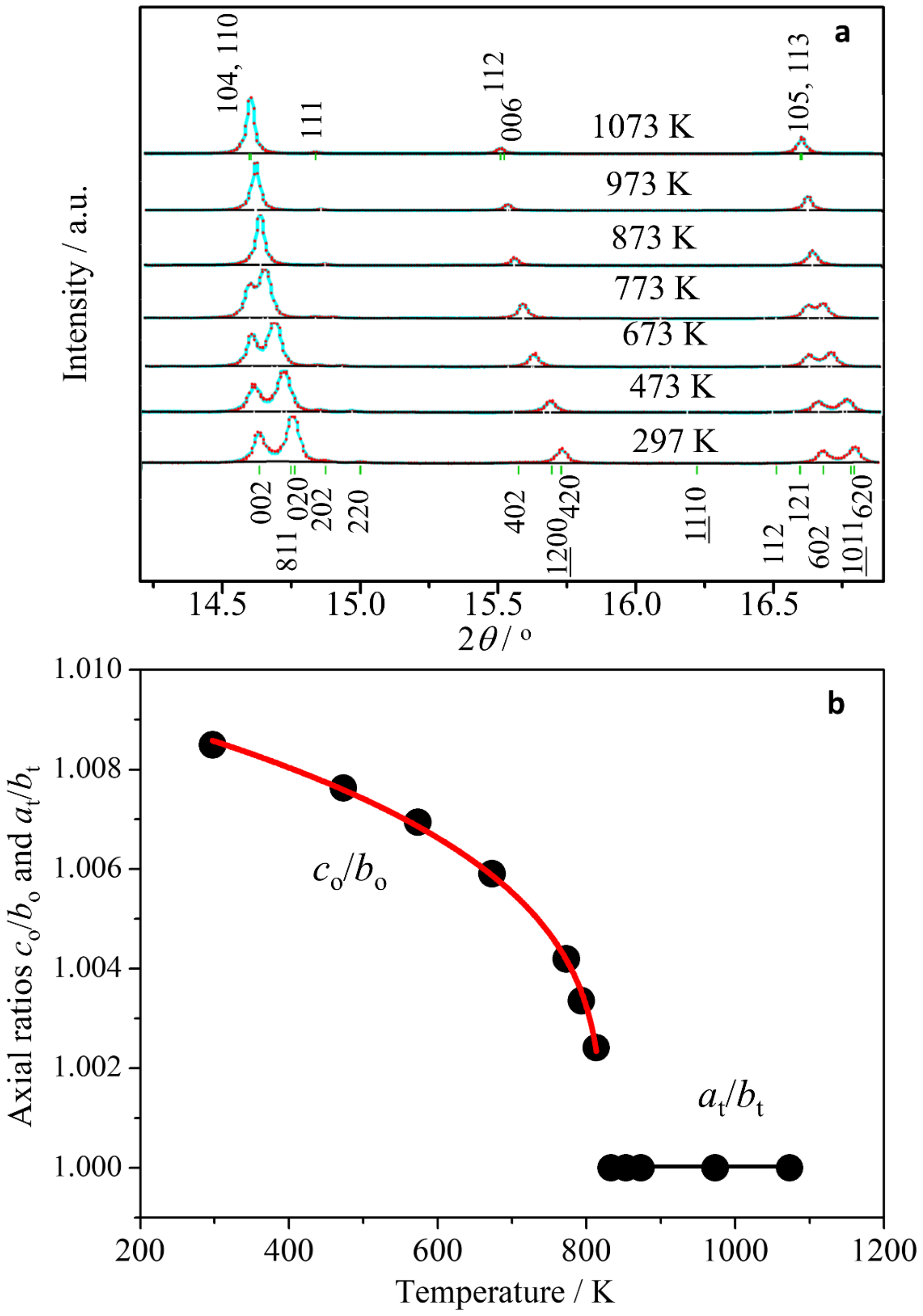

Supplementary Figure 3. Rietveld patterns and temperature dependence of axial ratio of $CsBi_2Ti_2NbO_{10-\delta}$ (**a**) Parts of Rietveld patterns of synchrotron X-ray powder diffraction data of $CsBi_2Ti_2NbO_{10-\delta}$ measured between 297 and 1073 K on heating. The *hkl* denotes the reflection index of the orthorhombic structure at 297 K and tetragonal structure at 1073 K. (**b**) Temperature dependence of axial ratios, c_o/b_o and a_t/b_t (= 1) of $CsBi_2Ti_2NbO_{10-\delta}$ during heating where the subscripts o and t denote the orthorhombic and tetragonal, respectively. Assuming a continuous phase transition, $c_o/b_o - 1 \propto (1 - T / T_C)^{\eta}$, the critical temperature T_C and critical exponent η were calculated to be 819.8(15) K and 0.298(10), respectively. The η was lower than that of a typical second-order phase transition of 0.5.[1]

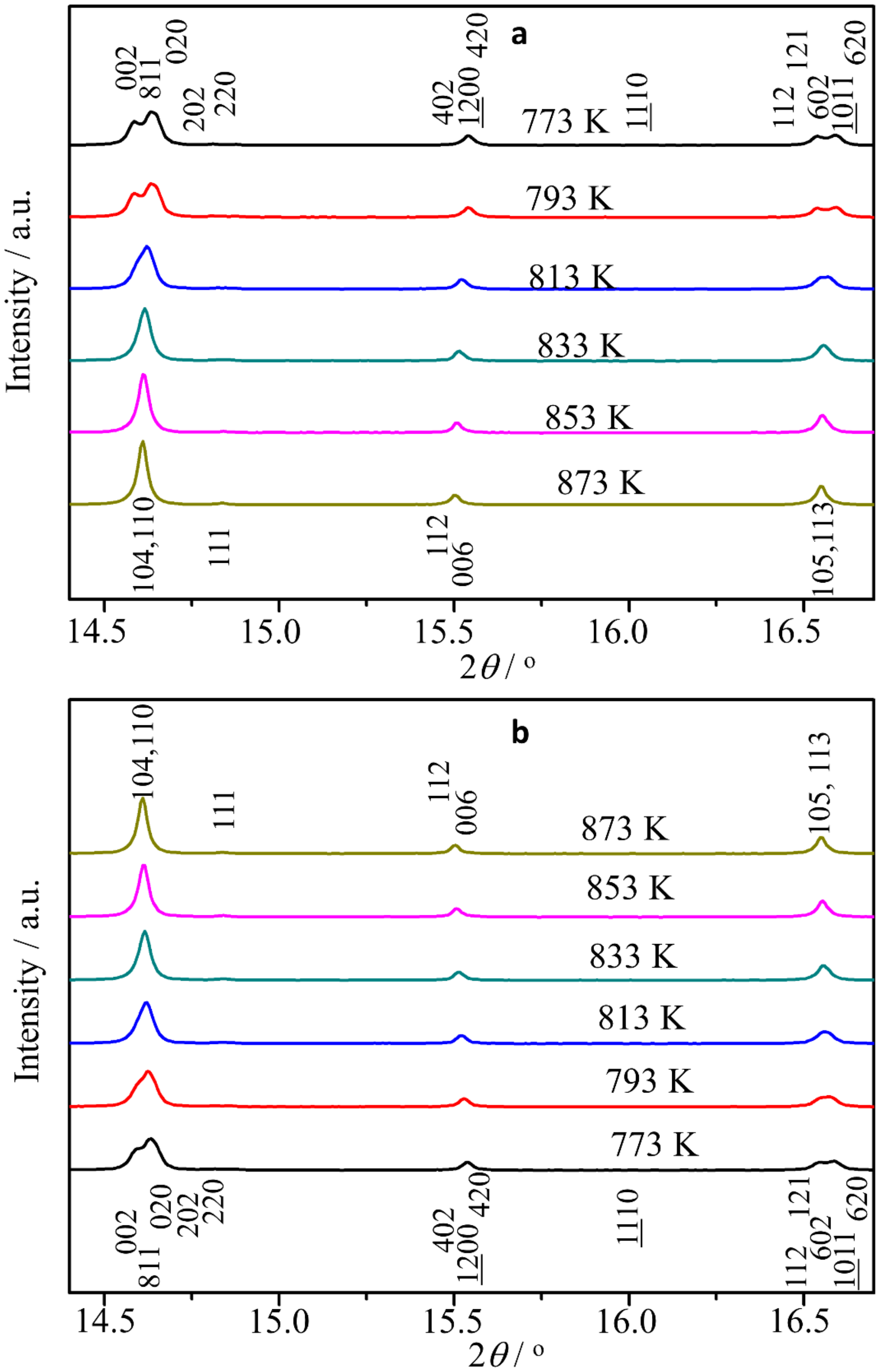

Supplementary Figure 4. Synchrotron X-ray diffraction patterns from 773 to 873 K on heating (a) and cooling (b) processes, showing a hysteresis of about 20 K.

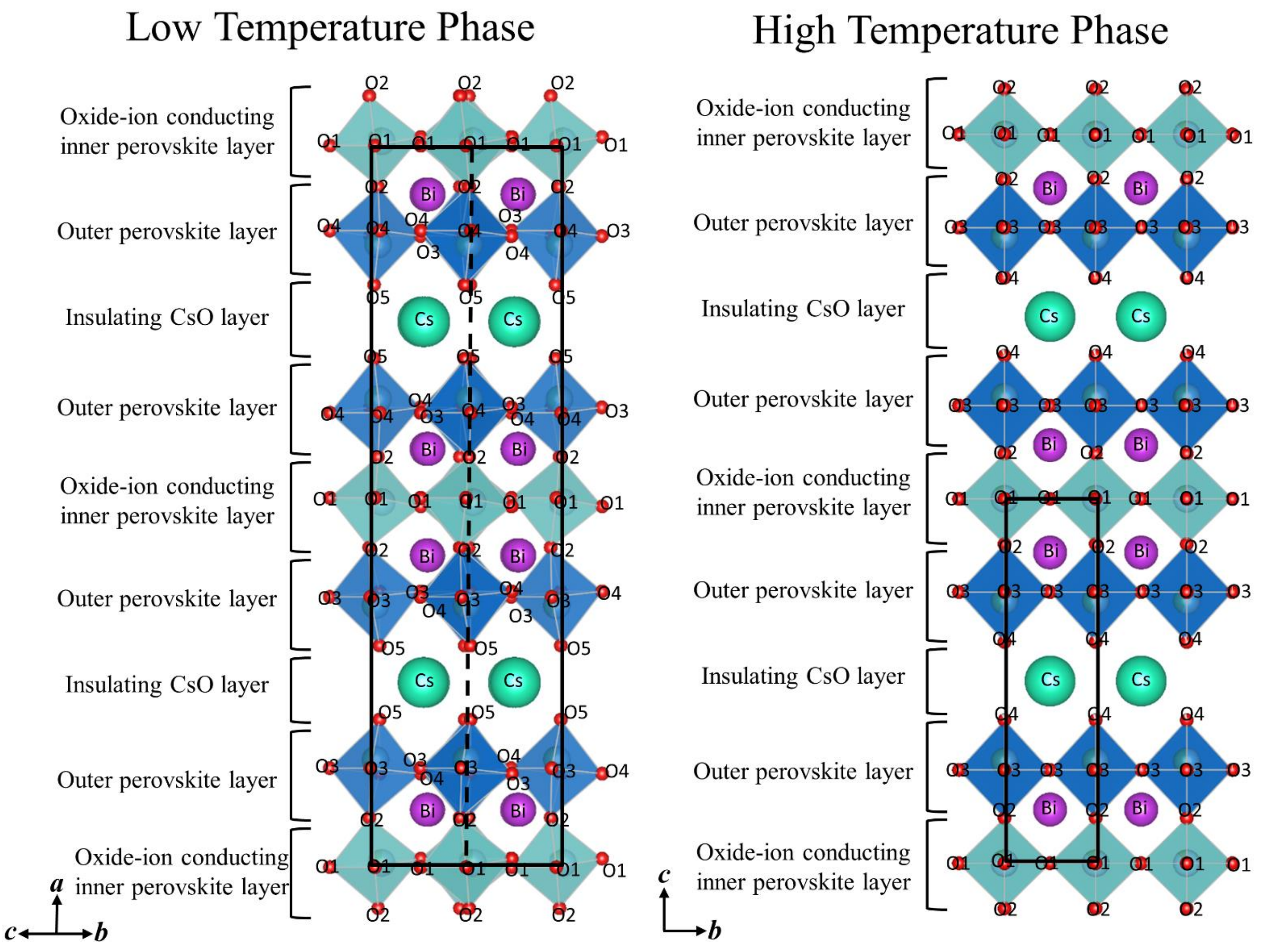

Supplementary Figure 5. Refined crystal structures of orthorhombic and tetragonal $CsBi_2Ti_2NbO_{10-\delta}$. Crystal structures of $CsBi_2Ti_2NbO_{10-\delta}$ refined using neutron-diffraction data: low-temperature orthorhombic phase (Left, 297 K, space group: *Ima*2) and high-temperature tetragonal phase (Right, 973 K, space group: *P*4/*mmm*). The low-temperature phase exhibits octahedral tilting and polar displacements.[2]

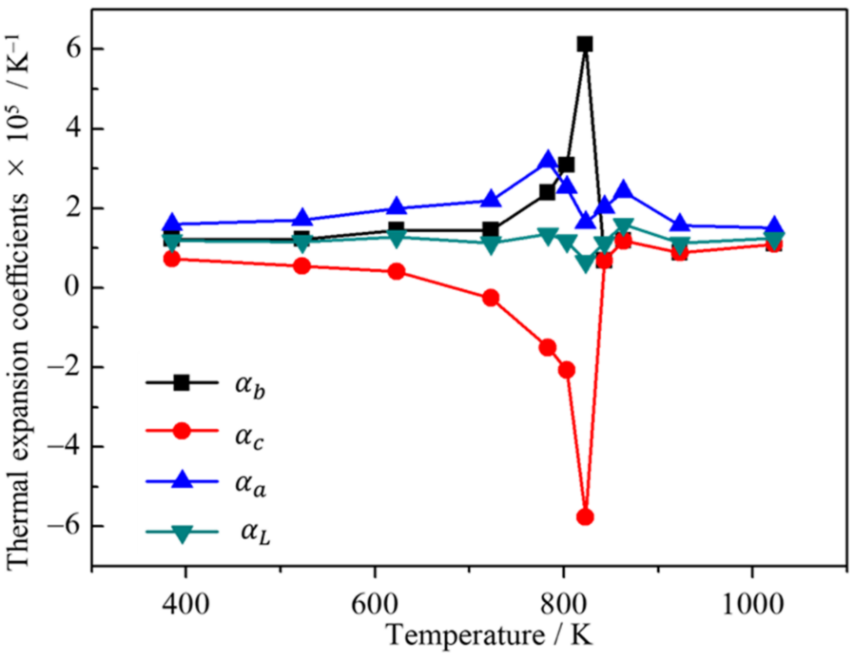

Supplementary Figure 6. Thermal expansion coefficients (TECs) of $CsBi_2Ti_2NbO_{10-\delta}$. The TECs were estimated using the lattice parameters refined by the Rietveld analyses of the synchrotron X-ray diffraction data (**Fig. 1a**). Thermal expansion anomalies were observed around the o-t phase transition temperature. TECs are defined as follows.

$\alpha_b = (b(T_{i+1}) - b(T_i)) / b(T_i) / (T_{i+1} - T_i)$ where $b(T_i) = b^{\mathrm{o}}(T_i)$ or $\sqrt{2} \cdot a^{\mathrm{t}}(T_i)$.

$\alpha_c = (c(T_{i+1}) - c(T_i)) / c(T_i) / (T_{i+1} - T_i)$ where $c(T_i) = c^{\mathrm{o}}(T_i)$ or $\sqrt{2} \cdot a^{\mathrm{t}}(T_i)$.

$\alpha_a = (a(T_{i+1}) - a(T_i)) / a(T_i) / (T_{i+1} - T_i)$ where $a(T_i) = a^{\mathrm{o}}(T_i)$ or $2 \cdot c^{\mathrm{t}}(T_i)$.

$\alpha_L = (v(T_{i+1}) - v(T_i)) / v(T_i) / (T_{i+1} - T_i)$ where $v(T_i) = [a^{\mathrm{o}}(T_i) \cdot b^{\mathrm{o}}(T_i) \cdot c^{\mathrm{o}}(T_i)]^{1/3}$ or $v(T_i) = [4 \cdot a^{\mathrm{t}}(T_i) \cdot a^{\mathrm{t}}(T_i) \cdot c^{\mathrm{t}}(T_i)]^{1/3}$.

Here, the i is a natural number between 1 and 11; T_1 = 297 K, T_2 = 473 K, T_3 = 573 K, T_4 = 673 K, T_5 = 773 K, T_6 = 793 K, and T_7 = 813 K (orthorhombic phase); T_8 = 833 K, T_9 = 853 K, T_{10} = 873 K, T_{11} = 973 K, and T_{12} = 1073 K (tetragonal phase).

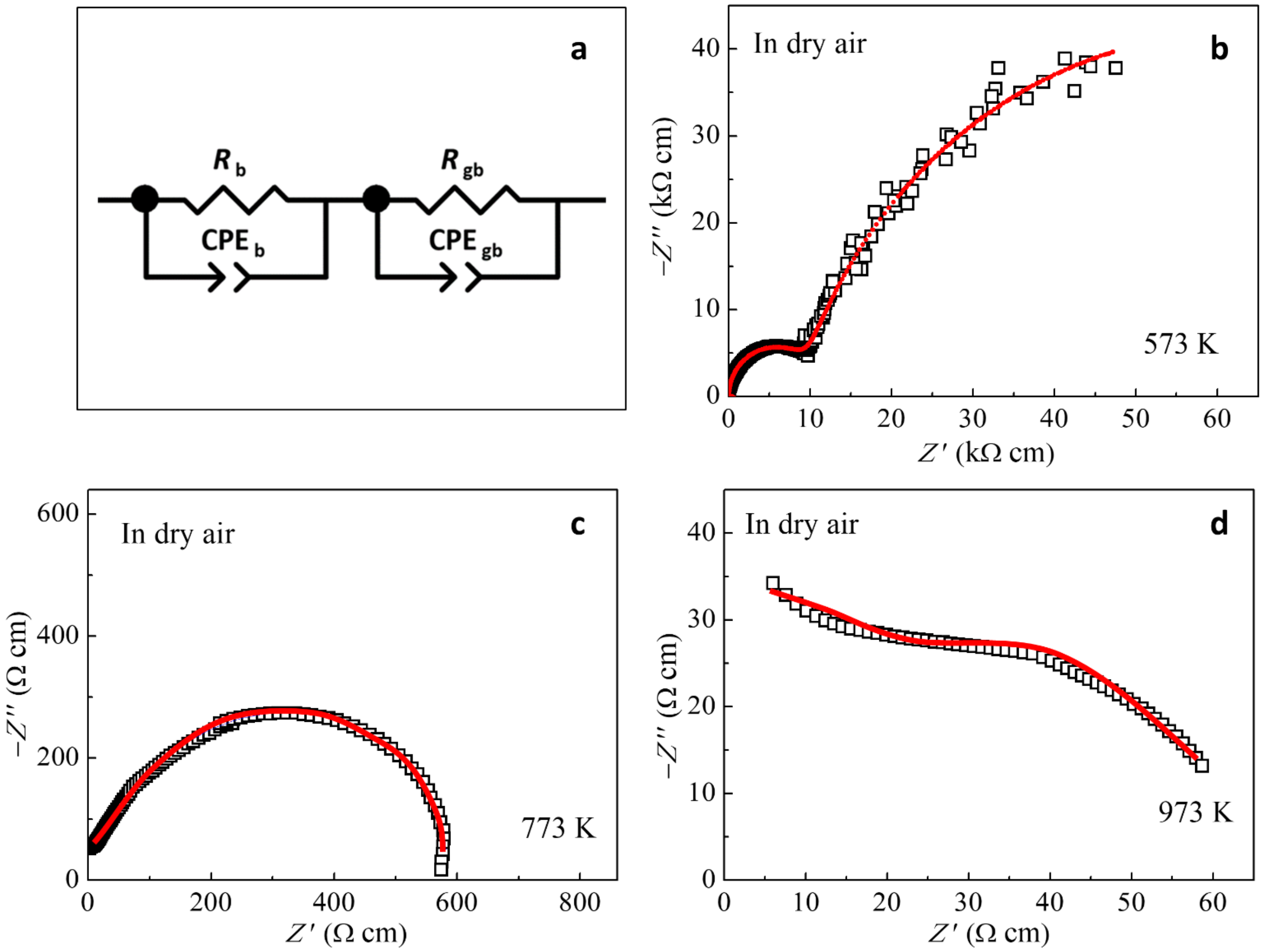

Supplementary Figure 7. Equivalent circuit and complex impedance plots recorded in flowing dry air. (**a**) Equivalent circuit used to fit the impedance spectroscopy data. *R* denotes a resistor and CPE stands for a constant phase element. The subscripts b and gb denote bulk and grain boundary, respectively. Complex impedance plots recorded in flowing dry air at (**b**) 573 K, (**c**) 773 K and (**d**) 973 K. Red line indicates the equivalent circuit fitting.

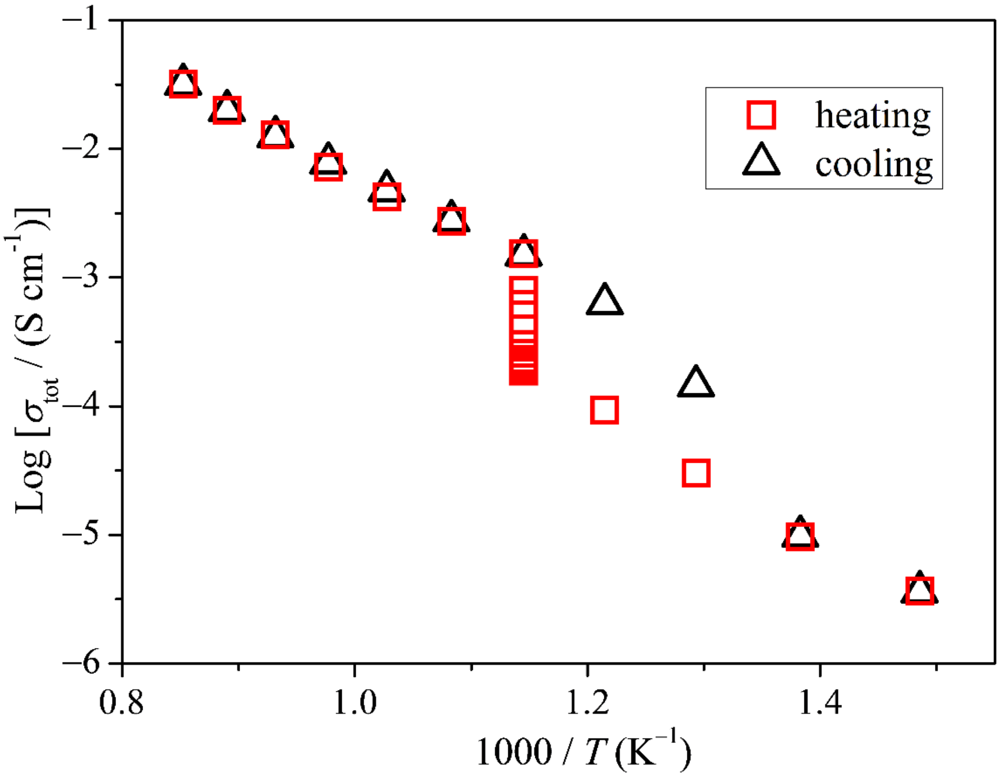

Supplementary Figure 8. Total DC electrical conductivities σ_{tot} of $CsBi_2Ti_2NbO_{10-\delta}$. The σ_{tot} from 673 to 1173 K on heating and cooling processes in static air, measured every 50 K and allowed 1 h of equilibration at each temperature. The conductivity abruptly increased by 16 times between 823 and 873 K on heating. The activation energy E_a for the σ_{tot} of high-temperature tetragonal phase 0.979(6) eV was lower than that of low-temperature orthorhombic one 1.093(8) eV

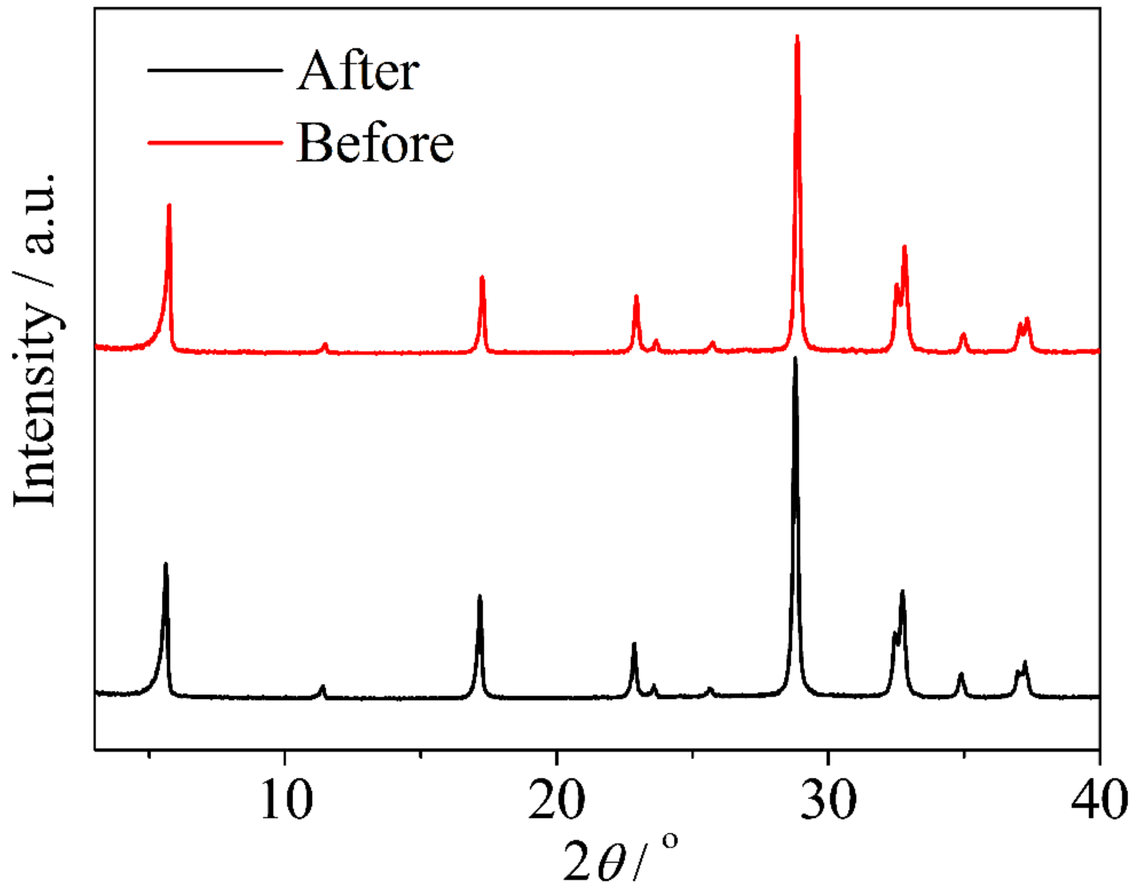

Supplementary Figure 9. X-ray powder diffraction patterns before and after impedance spectroscopy and oxygen concentration cell measurements.

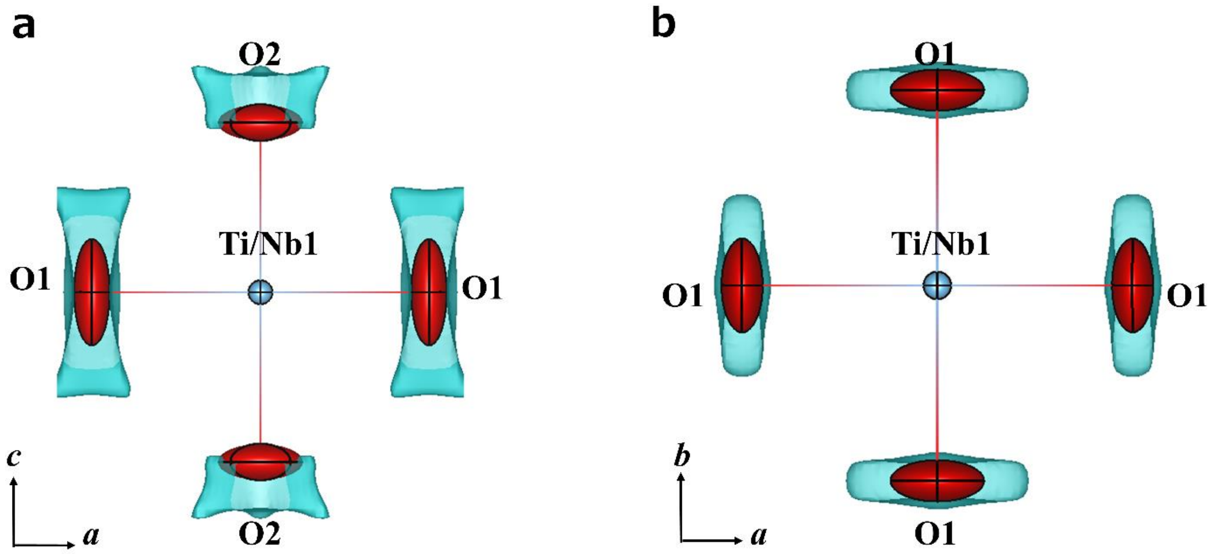

Supplementary Figure 10. Bond-valence-based energy landscapes (BVELs) for an oxide ion. BVEL along (a) the *b* and (b) *c* axes with the blue isosurface at 0.35 eV and red thermal ellipsoids for the refined crystal structure of $CsBi_2Ti_2NbO_{9.80(2)}$ at 973 K. Thermal ellipsoids are drawn at the 50% probability level. The BVELs are consistent with the shapes of thermal ellipsoids and anisotropic thermal motion of O1 and O2 atoms.

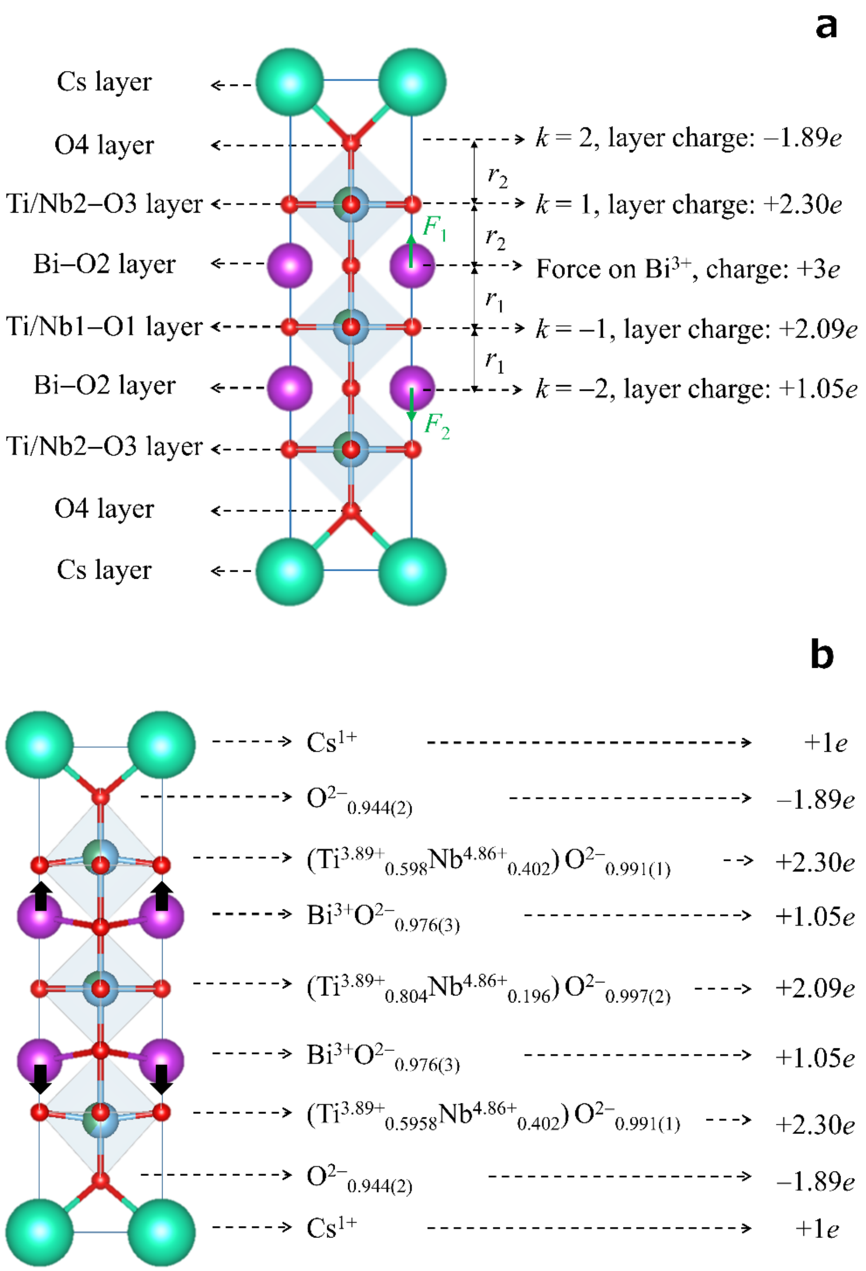

Supplementary Figure 11. Bi^{3+} displacement from the Bi–O2 layer due to electrostatic forces. (**a**) Hypothetical crystal structure of $CsBi_2Ti_2NbO_{10-\delta}$ without cation and anion displacements, consisting of Cs layers, O4 layers, Ti/Nb2–O3 layers, Bi–O2 layers and Ti/Nb1–O1 layer ($-1/2 \le x, y, z \le 1/2$). (**b**) Refined crystal structure of $CsBi_2Ti_2NbO_{9.80(2)}$, which was obtained using neutron-diffraction data at 973 K ($-1/2 \le x, y, z \le 1/2$). The r_1 stands for the distance between Bi^{3+} and Ti/Nb1–O1 layer, while the r_2 denotes the distance between Bi^{3+} and Ti/Nb2–O3 layer. F_1 and F_2 are the electrostatic forces on the Bi^{3+} cations at around z = 0.13 and –0.13, respectively. A value in (b) stands for the charge of each layer.

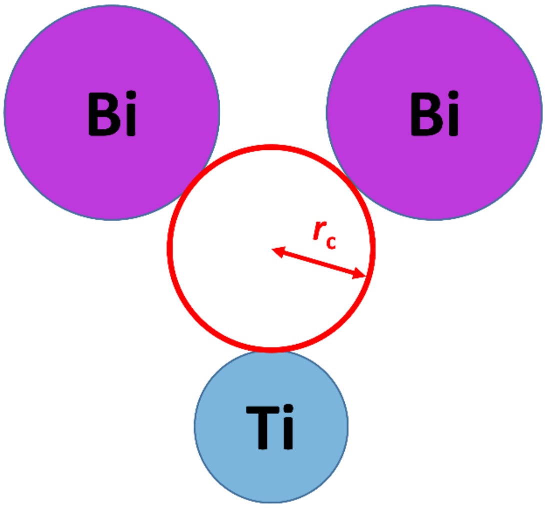

Supplementary Figure 12. Critical radius r_c for the Bi−Bi−Ti bottleneck triangle.

Supplementary Tables

Supplementary Table 1. Bond-valence-based energy barrier for oxide-ion migration (E_b)

No.	Composition	E_b (eV)	ICSD Code
1	$RbLaTa_2O_7$	0.83	86208
2	$RbLaTa_2O_7$	0.89	81870
3	$RbNdTa_2O_7$	0.55	258767
4	$RbLaNb_2O_7$	0.83	185293
5	$RbLaNb_2O_7$	0.90	185292
6	$RbCa_2Nb_3O_{10}$	0.51	260289
7	$RbCa_2Ta_3O_{10}$	0.45	89010
8	$RbSr_2Nb_3O_{10}$	0.79	93674
9	$RbLa_2Ti_2TaO_{10}$	0.74	93670
10	$RbLa_2Ti_2NbO_{10}$	0.90	90000
11	$RbBi_2Ti_2NbO_{10}$	0.48	252331
12	$RbPrNb_2O_7$	0.70	238794
13	$RbNdNb_2O_7$	0.65	258765
14	$Rb_{0.9}SrNb_2O_6F$	0.73	92867
15	$RbNaCa_2Nb_4O_{13}$	0.67	88853
16	$RbNa_{0.6}Sr_{0.4}Ca_2Nb_4O_{13}$	0.66	88854
17	$RbNa_{0.86}Ca_2Nb_3O_{10}$	0.59	97678
18	$CsCaLaTiNb_2O_{10}$	0.90	90624
19	$CsLa_2Ti_2NbO_{10}$	0.73	90623
20	$CsPr_2Ti_2NbO_{10}$	0.57	90625
21	$CsNd_2Ti_2NbO_{10}$	0.46	90626
22	$CsSm_2Ti_2NbO_{10}$	0.43	90627
23	$CsBi_2Ti_2NbO_{10}$	0.50	252331
24	$CsCa_2Ta_3O_{10}$	0.44	89011
25	$CsSrTa_2O_7$	0.72	93677
26	$CsNdTa_2O_7$	0.52	258766
27	$CsCa_2Nb_3O_{10}$	0.55	201425
28	$CsSr_2Nb_3O_{10}$	0.82	93675
29	$CsBa_2Nb_3O_{10}$	1.11	93676
30	$CsLaNb_2O_7$	0.84	82378
31	$CsNdNb_2O_7$	0.67	96511
32	$CsBiNb_2O_7$	0.55	162984
33	$CsLa_2Ti_2TaO_{10}$	0.80	93669
34	$CsBi_2Ti_{1.93}Ta_{1.07}O_{10}$	0.53	252332
35	$Cs_{1.09}PrNb_{2.06}O_7$	0.75	238796
36	$KCa_2Ta_3O_{10}$	0.49	89009
37	$KCa_2Nb_3O_{10}$	0.70	91098
38	$KCa_2Nb_3O_{10}$	0.55	157839

39	$KLaNb_2O_7$	0.90	72564
40	$KLaTa_2O_7$	0.90	83909
41	$LiLaTa_2O_7$	0.85	188780
42	$Li_{1.8}La_2VTi_3O_{11}$	0.90	171438
43	$LiCa_2Ta_3O_{10}$	0.46	89006
44	$LiLaNb_2O_7$	0.83	72566
45	$Li_{0.9}SrNb_2O_6$	0.75	92868
46	$LiLaTa_2O_7$	1.80	81872
47	$LiLaTa_2O_{6.14}N_{0.58}$	0.95	188782
48	$LiLaTa_2O_{6.74}N_{0.18}$	1.01	188781
49	$NaLaTa_2O_7$	0.90	84363
50	$NaCa_2Ta_3O_{10}$	0.51	88498
51	$NaCa_2Nb_3O_{10}$	0.51	97699
52	$NaLaNb_2O_7$	0.93	72565
53	$Na_{1.26}La_{0.913}Nb_2O_7$	0.87	67736
54	$NaLa_2Ti_2NbO_{10}$	0.90	51577
55	$NaPr_2Ti_2NbO_{10}$	0.60	51578
56	$NaPr_2Ti_2NbO_{10}$	0.50	51581
57	$NaNd_2Ti_2NbO_{10}$	0.50	51579
58	$NaSm_2Ti_2NbO_{10}$	0.45	51580
59	$AgLaNb_2O_7$	1.00	73983
60	$AgLaNb_2O_7$	0.85	73984
61	$AgLaTa_2O_7$	1.08	50165
62	$BaLa_2Ti_3O_{10}$	1.20	83749
63	$BaLa_2Ti_3O_{10}$	0.82	164868
64	$BaLa_2Ti_3O_{10}$	0.82	164869
65	$BaLa_2Ti_3O_{10}$	0.93	164870
66	$BaNd_2Ti_3O_{10}$	1.18	60955
67	$BaNd_2Ti_3O_{10}$	0.57	95516
68	$BaNd_2Ti_3O_{10}$	0.63	169675
69	$BaNd_2Ti_3O_{10}$	0.68	194985
70	$BaNd_2Ti_3O_{10}$	1.28	62217
71	$BaNd_2Ti_3O_{10}$	1.18	62218
72	$BaPr_2Ti_3O_{10}$	0.71	163911
73	$BaPr_2Ti_3O_{10}$	0.68	163912
74	$BaSm_2Ti_3O_{10}$	0.49	169676
75	$HLaNb_2O_7$	0.90	72567
76	$H_2Ca_2Nb_3O_{10.5}$	0.55	246002
77	$H_{0.76}Rb_{2.12}S_{0.76}LaNb_2O_7$	0.90	185290
78	$HRb_2SeLaNb_2O_7$	0.63	185291
79	$Br_{1.05}Ca_2CuNb_3O_{10}$	0.45	163235
80	$Br_{1.049}Ca_2Cu_{1.011}Ta_3O_{10}$	0.50	163238
81	$Br_{0.976}Sr_2Cu_{0.959}Nb_3O_{10}$	0.66	163237
82	$Br_{1.048}Sr_2Cu_{0.948}Ta_3O_{10}$	0.57	163236
83	$Cl_{1.13}Cu_{0.96}PrNb_{2.08}O_7$	0.75	238795

Supplementary Table 2. Crystallographic parameters of $CsBi_2Ti_2NbO_{9.97(1)}$ which were refined using neutron-diffraction data taken at 297 K with SuperHRPD diffractometer.

Site label *s*	Atom *X*	Oxidation number	$g(X; s)$[a]	*x*	*y*	*z*	U_{eq} (Å^2)[b]	BVS[c]
Cs	Cs	+1	1.000[d]	1/4	0.49941(4)	0.0012(4)	0.0126(4)	0.98
Bi	Bi	+3	0.992(2)	0.92575(3)	0.50806(18)	−0.0342(1)	0.0250(2)	2.97
Ti/Nb1	Ti	+ 4.18	0.822(2)[e]	0	0	−0.01774(13)	0.0059(1)	4.44
	Nb		0.178(2)[e]					
Ti/Nb2	Ti	+ 4.41	0.589(1)[e]	0.85896(2)	0.00575(18)	0.04243(18)	0.0034(2)	4.18
	Nb		0.411(1)[e]					
O1	O	−2	0.994(2)	0.0055(9)	0.75929(4)	0.28819(4)	0.0410(4)	2.04
O2	O	−2	1.0000[d]	0.937(5)	−0.05406(2)	0.05269(3)	0.0299(3)	1.99
O3	O	−2	1.0000[d]	0.87042(7)	0.77108(4)	0.30977(3)	0.0296(4)	2.08
O4	O	−2	0.998(2)	0.37499(5)	0.73288(3)	0.26039(3)	0.0085(3)	2.08
O5	O	−2	0.995(2)	0.80157(4)	0.02197(4)	0.01392(2)	0.0089(3)	1.75

Crystal system: orthorhombic; Space group: *Ima*2; Lattice parameters: a = 30.76478(10) Å, b = 5.450391(14) Å and c = 5.498837(14) Å, which are consistent with the literature.[2] Reliability factors: R_{wp} = 6.06%, R_p=4.48%, R_B = 6.33% and R_F = 5.99%.

[a] $g(X; s)$: Occupancy factor of *X* atom at the *s* site.

[b] U_{eq}: Equivalent isotropic atomic displacement parameter.

[c] Bond valence sums (BVSs) were calculated using bond valence parameters from references.[3,4]

[d] The occupancy factors of Cs, Bi and O atoms were refined in preliminary analyses. The occupancy factors of Cs, O3 and O4 sites were fixed to 1 because their refined occupancies were higher than 1

[e] The following linear constraints were used in the refinement: g(Ti; Ti/Nb1) + g(Nb; Ti/Nb1) = 1, g(Ti; Ti/Nb2) + g(Nb; Ti/Nb2) = 1, [g(Ti; Ti/Nb1) + g(Ti; Ti/Nb2)] : [g(Nb; Ti/Nb1) + g(Nb; Ti/Nb2)] = 2:1.

Supplementary Table 3. Crystallographic parameters of $CsBi_2Ti_2NbO_{9.80(2)}$ which were refined using neutron-diffraction data taken at 973 K *in situ* with SuperHRPD diffractometer.

Site label *s*	Atom *X*	Oxidation number	*g*(*X*; *s*)	*x*	*y*	*z*	U_{eq} (Å^2)	BVS
Cs	Cs	+1	1.000[a]	1/2	1/2	1/2	0.0472(8)	0.84
Bi	Bi	+3	1.000[a]	1/2	1/2	0.1485(1)	0.0789(5)	2.49
Ti/Nb1	Ti	+ 4.20	0.804(2)[b]	0	0	0	0.0075[d]	4.34
	Nb		0.196(2)[b]					
Ti/Nb2	Ti	+ 4.40	0.5978(11)[b]	0	0	0.2804(6)	0.0075[d]	4.31
	Nb		0.4022(11)[b]					
O1	O	–2	0.996(3)	0	1/2	0	0.0879(9)	1.84
O2	O	–2	0.976(3)	0	0	0.1257(1)	0.0734(8)	1.72
O3	O	–2	0.991(1)	0	1/2	0.2541(9)	0.0441(5)	1.99
O4	O	–2	0.944(3)	0	0	0.3926(1)	0.0382(5)	1.77

Site *s*	U_{11} (Å^2)[c]	U_{22} (Å^2)[c]	U_{33} (Å^2)[c]	U_{12} (Å^2)[c]	U_{13} (Å^2)[c]	U_{23} (Å^2)[c]
Cs	0.0248(9)	0.0248(9)	0.0918(3)	0	0	0
Bi	0.0650(6)	0.0650(6)	0.1068(3)	0	0	0
Ti/Nb1	0.0075[d]	0.0075[d]	0.0075[d]	0	0	0
Ti/Nb2	0.0075[d]	0.0075[d]	0.0075[d]	0	0	0
O1	0.0901(19)	0.0170(14)	0.157(3)	0	0	0
O2	0.1010(11)	0.1010(11)	0.0181(17)	0	0	0
O3	0.0100(4)	0.0100(4)	0.1123(18)	0	0	0
O4	0.0559(8)	0.0559(8)	0.0026(14)	0	0	0

Crystal system: tetragonal; Space group: *P*4/*mmm*; Lattice parameters: $a = b = 3.894032(7)$ Å and $c = 15.58224(6)$ Å, which are consistent with the literature.[2] However, M

Reliability factors: $R_{wp} = 2.39\%$, $R_p = 1.78\%$, $R_B = 4.05\%$, and $R_F = 8.01\%$.

[a] The occupancy factors of Cs, Bi and O atoms were refined in preliminary analyses. The occupancy factors were refined to be *g*(Cs; Cs) = 1.009(3), *g*(Bi; Bi) = 1.002(2), which indicates that *g*(Cs; Cs) = *g*(Bi; Bi) = 1.000 within 3 times of the estimated deviation.

[b] The following linear constraints were used in the refinement: *g*(Ti; Ti/Nb1) + *g*(Nb; Ti/Nb1) = 1, *g*(Ti; Ti/Nb2) + *g*(Nb; Ti/Nb2) = 1, [*g*(Ti; Ti/Nb1) + *g*(Ti; Ti/Nb2)] : [*g*(Nb;

Ti/Nb1) + g(Nb; Ti/Nb2)] = 2:1.

[c] U_{ij}: Anisotropic atomic displacement parameter.

[d] Atomic displacement parameters of Ti and Nb atoms were refined in preliminary analyses using the following linear constraints: U_{eq}(Ti/Nb1) = U_{eq}(Ti/Nb2) and fixed to the value of isotropic atomic displacement parameter (U_{iso}) in the final refinement.

Supplementary Table 4. Equivalent isotropic atomic displacement parameters (U_{eq}) and isotropic atomic displacement parameters (U_{iso}) of oxygen atoms refined using neutron and synchrotron X-ray powder diffraction data and the values in the literature.

	Site	U_{eq} refined using neutron diffraction data taken by SuperHRPD at J-PARC (973 K)	U_{iso} refined using synchrotron X-ray diffraction data at the beam line BL02B2 of SPring-8 (1073 K)	U_{iso} refined using neutron diffraction data in the literature[2] (973 K)
U_{eq} or U_{iso} (Å^2)	O1	0.0879(9)	0.087(2)	0.079(1)
	O2	0.0734(8)	0.088(3)	0.071(1)
	O3	0.0441(5)	0.045(1)	0.0302(6)
	O4	0.0382(5)	0.012(2)	0.038(1)

Supplementary Table 5. Comparisons of atomic displacement parameters of $CsBi_2Ti_2NbO_{10-\delta}$ with those of perovskite-type oxide-ion conductors.

Composition	$CsBi_2Ti_2NbO_{10-\delta}$			$La_{0.9}Sr_{0.1}Ga_{0.79}Mg_{0.21}O_{2.82}$[5]			$La_{0.8}Sr_{0.2}Ga_{0.8}Mg_{0.15}Co_{0.05}O_{2.8}$[6]		
Temperature	973 K			1023 K			1069 K		
U_{ij} (Å^2)	O1	U_{11}	0.0901(19)	O	U_{11}	0.059(3)	O	U_{11}	0.039(10)
		U_{22}	0.0170(14)		U_{22}	0.025(1)		U_{22}	0.018(8)
		U_{33}	0.1567(27)		U_{33}	0.072(1)		U_{33}	0.087(11)
	O2	U_{11}	0.1010(11)		U_{12}	–0.019(1)		U_{12}	0.018(8)
		U_{22}	0.1010(11)		U_{23}	0.011(2)		U_{23}	–0.012(3)
		U_{33}	0.0181(17)		U_{13}	0.016(2)		U_{13}	–0.012(3)
U_{eq} (Å^2)	O1	U_{eq}	0.0879(9)	O	U_{eq}	0.052(2)	O	U_{eq}	0.028(9)
	O2	U_{eq}	0.0734(8)						

Supplementary Table 6. Comparison of equivalent isotropic atomic displacement parameters of $CsBi_2Ti_2NbO_{10-\delta}$ with those of layered perovskites with Bi−Bi−Ti bottleneck triangles. The U_{eq}(O1) and U_{eq}(O2) of $CsBi_2Ti_2NbO_{10-\delta}$ at 973 K are much higher than equivalent isotropic atomic displacement parameters of other oxides.

Composition		$CsBi_2Ti_2NbO_{10-\delta}$				$BaBi_4Ti_4O_{15}$ [7]				$Bi_4Ti_3O_{12}$ [8]				$Bi_2Sr_2TiNb_2O_{12}$ [9]	
Temperature		RT		973 K		RT		1000K		RT		923 K		RT	
Space group		*Ima*2		*P*4/*mmm*		*A*2$_1$*am*		*I*4/*mmm*		*Aba*2		*Aba*2		*B*2*cb*	
U_{eq} (Å^2)	Oxide-ion conducting layer	O1	0.0410(4)	O1	0.0879(9)	O2	0.023(1)	O1	0.032(2)	O1	0.032(2)	O1	0.059(2)	O1	0.0106(5)
		O2	0.0299(3)	O2	0.0734(8)	O3	0.023(1)	O2	0.023(1)	O3	0.014(1)	O3	0.049(1)	O3	0.0228(5)
		O3	0.0296(4)			O4	0.014(1)	O3	0.023(1)	O5	0.023(1)	O5	0.038(2)	O4	0.0113(5)
						O7	0.020(2)	O4	0.014(1)	O6	0.034(2)	O6	0.036(2)	O5	0.009(1)
						O8	0.013(1)								
	other site	O4	0.0085(3)	O3	0.0441(5)	O1	0.032(2)	O5	0.018(1)	O2	0.007(1)	O2	0.030(1)	O2	0.0046(5)
		O5	0.0089(3)	O4	0.0382(5)	O5	0.018(1)	O6	0.0094(7)	O4	0.010(1)	O4	0.039(1)	O6	0.007(1)
						O6	0.0094(7)								

Supplementary Table 7. Comparison of bottleneck sizes (critical radii) and oxide-ion conductivities of $CsBi_2Ti_2NbO_{10-\delta}$ with those of layered perovskites with Bi−Bi−Ti bottleneck triangles. The oxide-ion conductivity of $CsBi_2Ti_2NbO_{10-\delta}$ is much higher than those of $BaBi_4Ti_4O_{15}$, $Bi_4Ti_3O_{12}$ and $Bi_2Sr_2TiNb_2O_{12}$.

Composition	$CsBi_2Ti_2NbO_{10-\delta}$	$BaBi_4Ti_4O_{15}$	$Bi_4Ti_3O_{12}$	$Bi_2Sr_2TiNb_2O_{12}$
References	This work	Ref. 10	Ref. 11	Ref. 12
Structure type	Dion-Jacobson	Aurivillius	Aurivillius	Aurivillius
Oxide-ion conductivity at 1073 K (S cm^{-1})	1.3×10^{-2} [a]	$< 4.0 \times 10^{-4}$ [b]	$< 1.0 \times 10^{-3}$ [b]	$< 1.3 \times 10^{-4}$ [b]
Critical radius (Å) [c]	1.0229(6)	0.9420(9)	0.9336(5)	0.9115(1)

[a] The oxide-ion conductivity σ_{ion} of $CsBi_2Ti_2NbO_{10-\delta}$ was estimated using the equation $\sigma_{ion} = \sigma_{tot} \times t_{ion}$ where the σ_{tot} is the total DC electrical conductivity and t_{ion} is the oxide-ion transport number.

[b] Total electrical conductivities σ_{tot} of $BaBi_4Ti_4O_{15}$, $Bi_4Ti_3O_{12}$ and $Bi_2Sr_2TiNb_2O_{12}$ are shown. The oxide-ion conductivity is lower than σ_{tot}, thus, we used the inequality "<" in the table as "$< 4.0 \times 10^{-4}$".

[c] The bottlenecks were evaluated by the critical radii r_c for the Bi−Bi−Ti bottleneck triangle (**Supplementary Figure 12**), which were calculated using the present room-temperature crystallographic data of $CsBi_2Ti_2NbO_{10-\delta}$ and those of $BaBi_4Ti_4O_{15}$, $Bi_4Ti_3O_{12}$ and $Bi_2Sr_2TiNb_2O_{12}$ reported in the refs 7, 8 and 9, respectively.

Supplementary Table 8. Average thermal expansion coefficients (TECs) of $CsBi_2Ti_2NbO_{10-\delta}$ in static air, which were estimated using the lattice parameters refined by the Rietveld analyses of the synchrotron X-ray diffraction data (Fig. 1a). The average TECs are anisotropic: $\boldsymbol{\bar{\alpha}_c^o} < \boldsymbol{\bar{\alpha}_b^o} < \boldsymbol{\bar{\alpha}_a^o}$ and $\boldsymbol{\bar{\alpha}_a^t} < \boldsymbol{\bar{\alpha}_b^t} < \boldsymbol{\bar{\alpha}_c^t}$. TEC values of $CsBi_2Ti_2NbO_{10-\delta}$ are similar with that of 8YSZ.[13]

Compositions	$CsBi_2Ti_2NbO_{10-\delta}$			8YSZ
Temperature range (K)	298–673	298–873	298–1073	RT–1273
$\bar{\alpha}_b^o$ or $\bar{\alpha}_b^t$ ($\times 10^{-6}$ K^{-1})	12.79	15.57	14.14	10.7
$\bar{\alpha}_c^o$ or $\bar{\alpha}_a^t$ ($\times 10^{-6}$ K^{-1})	5.93	0.81	3.16	
$\bar{\alpha}_a^o$ or $\bar{\alpha}_c^t$ ($\times 10^{-6}$ K^{-1})	17.36	19.27	18.32	
$\bar{\alpha}_L$ ($\times 10^{-6}$ K^{-1})	12.02	11.87	11.86	

The average TECs in the temperature range between T and T_0 are defined as follows.

$\bar{\alpha}_b^o = (b^o(T) - b^o(T_0)) / b^o(T_0) / (T - T_0)$, $\bar{\alpha}_b^t = (\sqrt{2} \cdot a^t(T) - b^o(T_0)) / b^o(T_0) / (T - T_0)$,

$\bar{\alpha}_c^o = (c^o(T) - c^o(T_0)) / c^o(T_0) / (T - T_0)$, $\bar{\alpha}_a^t = (\sqrt{2} \cdot a^t(T) - c^o(T_0)) / c^o(T_0) / (T - T_0)$,

$\bar{\alpha}_a^o = (a^o(T) - a^o(T_0)) / a^o(T_0) / (T - T_0)$, $\alpha_c^t = (2c^t(T) - a^o(T_0)) / a^o(T_0) / (T - T_0)$,

$\bar{\alpha}_L = (v(T) - v(T_0)) / v(T_0) / (T - T_0)$ where $v(T) = [a^o(T)\ b^o(T)\ c^o(T)]^{1/3}$ or $v(T) = [4a^t(T)\ a^t(T)\ c^t(T)]^{1/3}$

where T_0 = 298 K and the superscripts o and t denote the orthorhombic and tetragonal, respectively.

Supplementary Note 1. Calculation of the electrostatic force on a Bi^{3+} cation: Origin of the Bi^{3+} displacement

The Bi^{3+} cations are displaced apart from the Ti/Nb1–O1 layer. The displacement of a Bi^{3+} cation at z = 0.14851 along the c axis from the center of Ti/Nb1 site (z = 0) and Ti/Nb2 sites (z = 0.2804) was +0.129(2) Å. The Bi^{3+} displacement in $CsBi_2Ti_2NbO_{10-\delta}$ can be explained by the electrostatic forces as described below. The electrostatic force F between two points charges is expressed by the Coulomb's law:

$$F = \frac{q_1 q_2}{4\pi\varepsilon_0 r^2} \qquad (1)$$

where r is the distance between these two points charges q_1 and q_2, ε_0 is the permittivity of free space. To estimate the electrostatic force on a Bi^{3+} cation around z = 0.13, F_1, we consider the Coulomb's forces between the Bi^{3+} cation and four layers of k = ±2 and ±1 (**Supplementary Figure 11a**). The F_1 is calculated as follows,

$$F_1 = \frac{q_1 q_2}{4\pi\varepsilon_0 r^2} = \frac{3e}{4\pi\varepsilon_0} \times \left[-\frac{2.30e}{r_2{}^2} - \frac{-1.89}{(2r_2)^2} + \frac{2.09e}{r_1{}^2} + \frac{1.05}{(2r_1)^2}\right]$$

$$\approx \frac{3e}{4\pi\varepsilon_0} \times \left[-\frac{2.30e}{r_1{}^2} - \frac{-1.89e}{(2r_1)^2} + \frac{2.09e}{r_1{}^2} + \frac{1.05e}{(2r_1)^2}\right]$$

$$= \frac{3e}{4\pi\varepsilon_0} \times \frac{0.53e}{r_1{}^2} > 0 \qquad (2)$$

where the interatomic distance r_2 is assumed to be r_1. Thus, the Bi^{3+} cation around z = 0.13 can be displaced upward along the c axis (black arrows in **Supplementary Figure 11b**). Similarly, the electrostatic force on a Bi^{3+} cation around z = –0.13, F_2 is

$$F_2 \approx -\frac{3e}{4\pi\varepsilon_0} \times \frac{0.53e}{r_1{}^2} < 0 \qquad (3)$$

Thus, the Bi^{3+} cation around z = –0.13 can be displaced downward along the c axis (arrows in **Supplementary Figure 11b**). These results indicate that the Bi^{3+} cations in

$CsBi_2Ti_2NbO_{10-\delta}$ can be displaced apart from the Ti/Nb1–O1 (central Ti/Nb–O) layer due to the electrostatic forces. In n = 3 Dion-Jacobson phases A' [$A_{n-1}B_nO_{3n+1}$] (=A' [$A_2B_3O_{10}$], the A cation can be displaced apart from the central B–O layer due to the electrostatic forces which suggests that the n = 3 Dion-Jacobson phases could exhibit oxide-ion conduction.

Supplementary Methods

Computations: Screening of 69 candidates for oxide-ion conducting materials and investigation of the oxide-ion diffusion path by the bond-valence method.

We selected the chemical composition $CsBi_2Ti_2NbO_{10}$ as a candidate for oxide-ion conductors by screening 69 Dion–Jacobson phases using 83 crystallographic data through the bond-valence (BV) method.[14–18] Owing to its simple calculation procedure, the BV method is more efficient than the density functional theory (DFT)-based calculations and molecular dynamics (MD) simulations for exploring oxide-ion conductors. The bond-valence-based energy landscapes (BVELs) for a test oxide ion for 83 crystallographic data of 69 Dion–Jacobson phases were examined to search for oxide-ion conductors. The BV-based energy for each oxide was calculated using its crystallographic parameters from the inorganic crystal structure database (ICSD)[19] with the computer programme, softBV.[20] The BV parameters used in the calculations were given by Adams in the software.[21] The spatial resolution was set to 0.1 Å. The BV-based energy barriers, E_b, for the oxide-ion migration were estimated using BVELs (**Supplementary Figure 1**, **Supplementary Table 1)**. The E_b of $CsBi_2Ti_2NbO_{10}$ was relatively low (E_b = 0.5 eV). Furthermore, the BV-based energy of $CsBi_2Ti_2NbO_{10-\delta}$ was calculated with the crystallographic parameters refined using *in situ* high-temperature neutron-diffraction data taken at 973 K to investigate the anisotropic thermal motions and diffusion paths of the oxide ions.

Supplementary References

1. Yashima, M. & Ali, R. Structural phase transition and octahedral tilting in the calcium titanate perovskite $CaTiO_3$. *Solid State Ionics* **180**, 120–126 (2009).
2. McCabe, E. E. *et al.* Proper ferroelectricity in the Dion-Jacobson material $CsBi_2Ti_2NbO_{10}$: Experiment and theory. *Chem. Mater.* **27**, 8298–8309 (2015).
3. Krivovichev, S. V. Derivation of bond-valence parameters for some cation-oxygen pairs on the basis of empirical relationships between r_o and b. *Z. Kristallogr. Cryst. Mater.* **227**, 575–579 (2012).
4. Gagné, O. C. & Hawthorne, F. C. Comprehensive derivation of bond-valence parameters for ion pairs involving oxygen. *Acta Crystallogr. Sect. B Struct. Sci. Cryst. Eng. Mater.* **71**, 562–578 (2015).
5. Slater, P. R., Irvine, J. T. S., Ishihara, T. & Takita, Y. High-temperature powder neutron diffraction study of the oxide ion conductor $La_{0.9}Sr_{0.1}Ga_{0.8}Mg_{0.2}O_{2.85}$. *J. Solid State Chem.* **139**, 135–143 (1998).
6. Yashima, M. *et al.* Conduction path and disorder in the fast oxide-ion conductor $(La_{0.8}Sr_{0.2})(Ga_{0.8}Mg_{0.15}Co_{0.05})O_{2.8}$. *Chem. Phys. Lett.* **380**, 391–396 (2003).
7. Kennedy, B. J., Kubota, Y., Hunter, B. A., Ismunandar & Kato, K. Structural phase transitions in the layered bismuth oxide $BaBi_4Ti_4O_{15}$. *Solid State Commun.* **126**, 653–658 (2003).
8. Hervoches, C. H. & Lightfoot, P. A Variable-temperature powder neutron diffraction study of ferroelectric $Bi_4Ti_3O_{12}$. *Chem. Mater.* **11**, 3359–3364 (2002).
9. Surta, T. W. *et al.* Dielectric and ferroelectric properties in highly substituted $Bi_2Sr(A)TiNb_2O_{12}$ (A = Ca^{2+}, Sr^{2+}, Ba^{2+}) Aurivillius phases. *Chem. Mater.* **29**, 7774–

7784 (2017).

10. Thomas, J. K., Anderson, M. E., Krause, W. E. & zur Loye, H.-C. Oxygen ion conductivity in a new class of layered bismuth oxide compounds. *MRS Res. Soc. Symp. Proc.* **293**, 295–300 (1993).

11. Long, C., Fan, H., Ren, W. & Zhao, J. Double polarization hysteresis and dramatic influence of small compositional variations on the electrical properties in $Bi_4Ti_3O_{12}$ ceramics. *J. Eur. Ceram. Soc.* **39**, 4103–4112 (2019).

12. Shi, J. Ph.D. Dissertation, Crystal structure studies, electrical and magnetic properties of 2, 3, 4, 5-layer aurivillius oxides, Alfred University, Alfred, New York, 2015.

13. Vaßen, R., Kerkhoff, G. & Stöver, D. Development of a micromechanical life prediction model for plasma sprayed thermal barrier coatings. *Mater. Sci. Eng. A* **303**, 100–109 (2001).

14. Adams, S. Relationship between bond valence and bond softness of alkali halides and chalcogenides. *Acta Crystallogr. Sect. B Struct. Sci. Cryst. Eng. Mater.* **57**, 278–287 (2001).

15. Yashima, M. Invited Review: Some recent developments in the atomic-scale characterization of structural and transport properties of ceria-based catalysts and ionic conductors. *Catal. Today* **253**, 3–19 (2015).

16. Yasui, Y., Niwa, E., Matsui, M., Fujii, K. & Yashima, M. Discovery of a rare-earth-free oxide-ion conductor $Ca_3Ga_4O_9$ by screening through bond valence-based energy calculations, synthesis, and characterization of structural and transport properties. *Inorg. Chem.* **58**, 9460-9468 (2019).

17. Niwa, E. & Yashima, M. Discovery of oxide-ion conductors with a new crystal

structure, $BaSc_{2-x}A_xSi_3O_{10-x/2}$ (A = Mg, Ca) by screening sc-containing oxides through the bond-valence method and experiments. *ACS Appl. Energy Mater.* **1**, 4009–4015 (2018).

18. Avdeev, M., Sale, M., Adams, S. & Rao, R. P. Screening of the alkali-metal ion containing materials from the Inorganic Crystal Structure Database (ICSD) for high ionic conductivity pathways using the bond valence method. *Solid State Ionics* **225**, 43–46 (2012).

19. Belkly, A., Helderman, M., Karen, V. L. & Ulkch, P. New developments in the Inorganic Crystal Structure Database (ICSD): Accessibility in support of materials research and design. *Acta Crystallogr. Sect. B Struct. Sci. Cryst. Eng. Mater.* **58**, 364–369 (2002).

20. Chen, H., Wong, L. L. & Adams, S. SoftBV – a software tool for screening the materials genome of inorganic fast ion conductors. *Acta Crystallogr. Sect. B Struct. Sci. Cryst. Eng. Mater.* **75**, 18–33 (2019).

21. Chen, H. & Adams, S. Bond softness sensitive bond-valence parameters for crystal structure plausibility tests. *IUCrJ* **4**, 614–625 (2017).
